# Re-irradiation for head and neck cancer: outcome and toxicity analysis using a prospective single institution database

**DOI:** 10.3389/fonc.2023.1175609

**Published:** 2023-06-29

**Authors:** Chiara Scolari, André Buchali, Achim Franzen, Robert Förster, Paul Windisch, Stephan Bodis, Daniel R. Zwahlen, Christina Schröder

**Affiliations:** ^1^ Department of Radiation Oncology, University Hospital Ruppin-Brandenburg, Brandenburg Medical School Theodor Fontane (MHB), Neuruppin, Germany; ^2^ Department of Radiation Oncology, Cantonal Hospital Winterthur (KSW), Winterthur, Switzerland; ^3^ Department of Otorhinolaryngology-Head and Neck Surgery, University Hospital Ruppin-Brandenburg, Brandenburg Medical School Theodor Fontane (MHB), Neuruppin, Germany; ^4^ Faculty of Health Sciences Brandenburg, Joint Faculty of the University of Potsdam, Brandenburg university of Technology Cottbus-Senftenberg and Brandenburg Medical School, Potsdam, Germany; ^5^ Center for Radiation Oncology, Cantonal Hospital Aarau and Baden (KSA-KSB), Aarau/Baden, Switzerland

**Keywords:** head and neck cancer, loco-regionally recurrence, second primary, re-irradiation, toxicity, survival, locoregional control

## Abstract

**Purpose:**

Re-irradiation (re-RT) in head and neck cancer is challenging. This study prospectively explored the feasibility of re-RT in patients with loco-regionally recurrent or second primary head and neck cancer (LRR/SP HNC).

**Methods:**

From 2004 to 2021, 61 LRR/SP HNC patients were treated with re-RT, defined as having a second course of RT with curative intent resulting in a cumulative dose of ≥100 Gy in an overlapping volume. Postoperative or definitive dynamic intensity-modulated and/or volumetric modulated re-RT was administered using twice daily hyperfractionation to 60 Gy combined with cisplatin or carboplatin/5-fluorouracil. Overall survival (OS), progression-free survival (PFS), locoregional control (LRC) and distant metastasis control (DMC) were analyzed and prognostic factors evaluated. Toxicity was prospectively recorded and graded.

**Results:**

The median follow-up was 9.8 months. In 41 patients (67.1%), complete administration of the intended treatment was not feasible. In 9 patients (15%) re-RT was interrupted prematurely and in other 9, the complete re-RT dose was lower than 60 Gy, and 37 patients (61%) could not receive or complete chemotherapy. Two-year OS, PFS and LRC rates were 19%, 18% and 30%, respectively. 20 patients (33%) received the complete intended treatment, and 1- and 2-year OS rates were 70% and 47%, respectively. Charlson comorbidity index was an important predictor for treatment completion. Multivariate analysis revealed recurrent N stage 0–1, age, chemotherapy administration and re-RT dose of 60 Gy as prognostic factors for clinical outcomes. No grade 5 re-RT-related toxicity was observed. The most common new grade ≥3 acute toxicities were dysphagia (52%) and mucositis (46%). Late toxicity included grade ≥3 dysphagia in 5% and osteoradionecrosis in 10% of evaluable patients, respectively. 6 patients (10%) were alive after 9 years without progression and no late toxicity grade ≥3, except for 2 patients presenting with osteoradionecrosis.

**Conclusion:**

Hyperfractionated re-RT with 60 Gy combined with platinum-based chemotherapy was a curative treatment option with acceptable toxicity in LRR/SP patients. Patients with higher comorbidity had a higher probability of failing to receive and complete the intended therapy. Consequently, they derived unsatisfactory benefits from re-RT, highlighting the importance of patient selection.

## 1 Introduction

Despite advances in the multimodality treatment of head and neck cancer (HNC), locoregional recurrence (LRR) or second primary tumors (SP) within or in close proximity to a previously irradiated area remains a common and challenging clinical scenario and represents the most frequent cause of death (1, 2). Five years after treatment, LRR HNC can occur in 16%–25% (1, 3) and 17%–52% (3, 4) of patients treated with postoperative and definitive chemoradiation, respectively. Approximately 4%–15% will develop a SP cancer within 5 years of definitive RT for HNC, and 25%–30% of these are in the head and neck region (4, 5). The risk of SP cancer increases with time, with a 15-year rate of 25% (4). Salvage surgery is still considered the primary curative treatment option, although in many patients with LRR/SP HNC it is not possible due to macro- and microscopically unresectable tumor, medical comorbidities or patient refusal. In the literature, 2-year OS rate between 30–50% after salvage surgery in LRR HNC patients were reported (6). Oncologic risk factors for reduced outcomes were advanced primary tumor and nodal stage, short disease-free interval, non-laryngeal cancer site and previous RT (6). In SP HNC patients, the outcomes after salvage surgery seem to be better, with a 3-year recurrence-free survival rate of 40% (versus 17% in LRR HNC patients), although at 5 years the rate of the two clinical scenarios became similar (7). In other studies, survival rates for LRR versus SP seem similar (8).

Nowadays, postoperative or definitive (chemo)-re-irradiation (re-RT) is considered another potentially curative treatment option in well-selected patients with LRR/SP HNC (9). Although historically re-RT was associated with significant acute and late toxicity and a relatively poor chance of long-term cure (10–12), several studies have demonstrated an improvement in therapeutic ratio with modern treatment techniques such as intensity-modulated radiotherapy (IMRT) that allows more conformal and targeted higher dose delivery while minimizing normal tissue toxicity and improving tumor control (13–18). Nevertheless, even among recent studies using IMRT, the risk of severe late toxicity in long-term survivors remains significant and a wide range of 2-year OS rates from 17% to 76% have been reported, depending on patient selection criteria and different cohort characteristics (8, 19–27). Lower 2-year OS rate (29%) was observed in one recent analysis excluding patients treated with surgery (27), as well as non-squamous cell carcinoma and nasopharyngeal, sinonasal, base of skull and other non-laryngopharyngeal tumors. The inclusion of both squamous and non-squamous cell carcinoma may have contributed to obtain higher 2-year OS rates (52%–76%) (23–25), compared to other analysis in whom non squamous cell carcinoma were excluded (8). The inclusion of SP HNC, and a higher proportion of these in the study cohort, may have similarly led to better results (24) than in studies in which SP HNCs were excluded or the proportion was lower (19). Results also depend on the application of different re-RT definitions, as shown by a recent study, that used a strict definition of re-RT, resulting in a 2-year OS rate of 42.6%, lower than in other analyses (28). Moreover, in one study reporting a 2-year OS rate of 51%, patients who discontinued treatment were excluded (20). This exclusion criteria may have led to a favorable effect on the survival outcomes. In addition to these different patient selection criteria, differences in the distribution of patients (comorbidity, organ dysfunction, age), tumor (T and N stage, localization, disease-free interval, presentation type) contribute to further enlarge the observed range of 2-year OS rates, suggesting that re-RT may be beneficial only in a well-selected cohort of patients (8, 20, 29–34). Several studies validated a recursive partitioning analysis (RPA) classification to facilitate the identification of ideal candidates for re-RT (8, 35, 36). The 2-year OS rates were 62% and 17% in the most and least favorable RPA class, respectively (8). However, further data are needed to improve decisions on benefit-risk balance. In particular, there is a scarcity of recent studies reporting prospectively collected toxicity of re-RT for HNC. An updated large systematic review on re-RT in HNC patients identified 223 retrospective analyses in this field versus 23 prospective studies, of which only two were published less than a decade ago with a very small number of patients included (9, 37, 38). This study, therefore, aimed to offer added value to the literature by analyzing prospectively acquired data of LRR/SP HNC patients in whom the same treatment protocol and techniques were used: IMRT and/or volumetric modulated re-RT using twice daily hyperfractionation to a total dose of 60 Gy combined with cisplatin or carboplatin plus 5-fluorouracil.

## 2 Materials and methods

### 2.1 Patients selection

We screened and analyzed prospectively acquired data of all patients who received hyperfractionated intensity-modulated and/or volumetric modulated re-RT for loco-regionally recurrent (LRR) or second primary (SP) HNC at the University Hospital Ruppin-Brandenburg from 07.2004 to 12.2021 (n=68). Re-RT was defined as a second course of RT with curative intent (dose >50 Gy) in a region previously irradiated with a curative dose of ≥56 Gy, resulting in an overlapping volume with a cumulative dose of ≥100 Gy in a biologically equivalent dose of 2 Gy per fraction (EQD2, obtained using the linear-quadratic model and α/β=3). For patients receiving their first course of RT before 2006 or outside the institution, and for whom dose plans were therefore not available electronically (n=15), the overlapping volume was estimated by visually comparing the treatment plans of both the first and second treatment. All LRR/SP HNCs had histological confirmation with a biopsy. SP HNCs were defined as tumors of different histologies, different sites of origin or the same site but occurring >5 years after diagnosis of previous HNC. Comorbidity was evaluated at the time of retreatment and was measured using the Charlson comorbidity index. Patients initially diagnosed with distant metastasis (n=4), presenting large cell carcinoma or adenocarcinoma (n=2) or in whom the pattern of failure was unknown (n=1) were excluded. Finally, 61 patients were eligible for this study. This analysis was approved by the Ethics Committee of the Brandenburg Medical School “Theodor Fontane” (MHB) (E-01-20220110, approval date 25.01.2022).

### 2.2 Treatment characteristics

Patients with LRR/SP HNC were evaluated by a multidisciplinary tumor board. If possible, surgery was performed. In the surgical decision making, factors such as operability with regard to the general surgical risks, chance of R0 resection and possible postoperative complications (swallowing function, voice, breathing) were considered. At this institution, the chemotherapy regimen and the dose/fractionation schedule for re-RT in LRR/SP patients remained the same over the time frame in which data were collected for the present analysis. In general, all patients were offered concurrent and adjuvant chemotherapy using cisplatin (Cis, 75 mg/m²/day, d 1) plus 5-fluorouracil (5FU, 800 mg/m² KOF/day, d 1–5) administered in three or four cycles (two cycles simultaneously at week 1 and 4 and one or two in the adjuvant setting). In the case of reduced creatinine clearance (GFR <90 ml/min), carboplatin (Carbo, AUC 5, day 1) plus 5-fluorouracil (5FU, 800 mg/m² KOF/day, d 1–5) was given. If there were contraindications for the use of platinum-based agents, Cetuximab was administered instead (400 mg/m² one week before re-RT and 250 mg/m² weekly during re-RT). Systemic therapy was contraindicated and, therefore, not administered in cases of GFR <40 ml/min, age >75 years, patient refusal, poor general condition (ECOG ≤2) or significant comorbidity. No induction chemotherapy was administered. Before re-RT, all patients underwent a computed tomography (CT) based simulation using intravenous contrast media and were immobilized with a thermoplastic mask to allow reproducibility of treatment setup and positioning during re-RT. Most of the first RT and all re-RT were performed using dynamic intensity-modulated radiotherapy (IMRT) and/or volumetric modulated radio-therapy (VMAT) which were introduced at the University Hospital Ruppin-Brandenburg in 2002 and 2010, respectively. Neither image nor stereotactic guidance was used, except in four patients treated after the recent introduction of image-guided RT (without 6D treatment couches). The gross tumor volume (GTV) was defined as all visible disease in treatment planning CT or MRI. A 6 mm expansion around the GTV created the clinical target volume (CTV). In postoperative patients, the CTV was defined according to the visible tumor bed and the preoperative diagnostics. The planning target volume margin (PTV) was typically 6 mm or, in the patients who received image-guided RT, reduced to 3 mm. In general, the dose prescribed to the PTV was 60 Gy, administered using hyperfractionation (1.2 Gy per fraction, twice daily, at least 8 hours apart, 5 days per week). In some cases, for reasons related to organs at risk, the total prescribed dose was lower than 60 Gy (>50 Gy and <60 Gy). Elective neck irradiation was generally avoided. The planned maximum dose (D_max_) from each course of RT was converted to an equivalent dose in 2−Gy fractions (EQD2) using α/β=2. The cumulative maximum point dose constraint to the optic chiasm/nerve was an EQD2 of 50 Gy. For the spinal cord and the brainstem, a certain degree of repair of sublethal damage was taken into account. Therefore, the dose constraints to these two organs at risk depended on the interval between the date of the first RT and the re-RT. If the time interval was less than six months, the cumulative maximum point dose to the spinal cord could not exceed an EQD2 of 50 Gy and the brainstem an EQD2 of 55 Gy. If the time interval was more than six months, the cumulative maximum point dose to both the spinal cord and the brainstem could not exceed an EQD2 of 60. No other specific dose constraints were used other than to limit the dose to salivary glands, mandible, pharyngeal constrictor muscles and other designated structures at risk as much as possible. RT was planned using the EclipseTM treatment planning system (Varian Medical Systems, Palo Alto, CA, USA) and delivered with 6-MV photon linear accelerators Varian® (Palo Alto, CA, USA).

### 2.3 Statistical analysis

Overall survival (OS), locoregional control (LRC), distant metastasis control (DMC) and progression-free survival (PFS) were analyzed. The following definitions of events were used: death from any cause (OS), locoregional recurrence independent of its correlation to the radiation fields (LRC), distant metastasis (DMC), tumor progression or death of any cause, whichever came first (PFS). All time-to-event analyses were calculated from the start date of re-RT to the date of each event of interest or the last follow-up according to the Kaplan-Meier method. Univariate and multivariate analysis using Cox proportional hazard models were performed to evaluate potential prognostic factors for OS, PFS, LRC and DMC, including recurrent T stage (dichotomized as 4 vs. 0–3), recurrent N stage (2–3 vs. 0–1), disease-free interval (≤24 vs. >24 months), age at the start of re-RT (>60 vs. ≤60 years), Charlson comorbidity index (≥3 vs. 1–2), baseline dysphagia (grade 3–4 vs. 0–2), surgery (yes vs. no), chemotherapy (indicated but not administered vs. early terminated vs. administered as planned) and re-RT dose (60 vs. <60 Gy). Dichotomization of the prognostic factors was performed on the basis of cutoff values provided by the current literature (8, 19, 20, 33, 35). Patients who received Cetuximab (n=3) and one patient without indication for chemotherapy were excluded from the evaluation of chemotherapy as a prognostic factor in the univariate and multivariate analysis. Spearman’s rank correlation test was performed to avoid including in the multivariate analysis strongly correlated factors (we defined a correlation coefficient of 0.8 as the cut-off value). Univariate and multivariable logistic regression analyses of predictors of treatment completion were performed. Completion of therapy was defined as receiving re-RT as intended (with 60 Gy) plus chemotherapy (early terminated or completed as planned). Univariate logistic regression analyses were also employed to evaluate the relationship between various variables; in the case of non-dichotomous variable, chi-square test was performed. Patients were additionally divided into three recursive partitioning analysis (RPA) classes in analogy to the definition by the Multi-Institution Reirradiation (MIRI) Collaborative: class I included patients >2 years from the first course of RT with resected tumors regardless of margin status; class II included patients >2 years with unresected tumors or ≤2 years with baseline dysphagia grade 0–2; class III included patients ≤2 years from the initial course of RT with baseline dysphagia grade 3–4 (8). We defined baseline organ dysfunction as grade 3–4 dysphagia. Survival outcomes of each subgroup were analyzed. Patients were followed up at least every three months for two years, then every six months for three years and annually thereafter. In case of suspicion of recurrence, MRI or CT was performed and, whenever possible, histologically confirmed.

Toxicity was prospectively collected according to the National Cancer Institute’s Common Terminology Criteria for Adverse Events (CTCAE), whose versions over the years have not changed substantially in terms of grading the adverse events reported in the present study. For the evaluation of fibrosis and telangiectasia, the Late Effects Normal Tissue Task Force (LENT)-Subjective, Objective, Management, Analytic (SOMA) scale was used instead. Acute toxicity was defined as the highest grade of toxicity occurring during or within 90 days from re-RT completion. Any adverse event developing or persisting 90 days beyond the end of re-RT was considered late toxicity. To avoid accounting for morbidities resulting from prior treatments, disease progression or further therapy after re-RT, toxicities already present prior to the re-RT were considered and adverse events were censored on tumor progression or on further treatment that could have influenced the toxicity. A P value of <.05 was considered to be statistically significant. Statistical analyses were performed with IBM SPSS statistics 28 (Statistical Package for Social Sciences, International Business Machines Corp., Armonk, NY).

## 3 Results

### 3.1 Patients and tumors characteristics

**Table 1** and **Supplementary Table 1** show the characteristics of the HNC patients treated with the first RT and re-RT, respectively. The median age of the study population at initial radiotherapy (RT) was 56.5 years (interquartile range [IQR], 48.1–62.2) and at re-RT was 59.8 years (IQR, 53.1–66.7). Most patients were male (91.8%) and 21 patients (34.4%) developed SP HNC. Twenty-five patients (41.0%) presented a Charlson comorbidity index ≥3, and a history of myocardial infarction was found in 14 patients (23%; **Table 1** and **Supplementary Table 2**). The most common subsite of disease recurrence was the oropharynx (29.5%), followed by the tongue and floor of the mouth (26.2%). Lymph node-only recurrence accounted for 16.4% (n=10). The majority of SP HNCs (85.7%) was localized in oropharynx (n=8), tongue and floor of the mouth (n=8) and hypopharynx (n=2) (**Supplementary Table 3**). In all patients the histology of LRR/SP HNC was squamous cell carcinoma. Thirty-one (50.8%) patients showed T4 stage. T stage distribution was different between patients with Charlson comorbidity index ≥3 (T 4 stage in 28% of the cases) and those with an index of 1–2 (T4 stage in 66.7% of the cases; p=0.004; **Supplementary Tables 4**). All laryngeal tumors (n=8) and two of the three nasopharyngeal tumors presented T4 stage (**Supplementary Table 5**). 46% of patients had lymph node involvement. For 13 patients (21.3%), the recurrence treated with re-RT was not the first one. Nineteen patients (31.1%) exhibited baseline grade 3–4 dysphagia, most of which (78.9%) with a disease-free interval of ≤24 months (vs. 47.6% of patients with baseline grade 0–2 dysphagia).

**Table 1:** Patient and tumor characteristics at the re-irradiation (n=61).

		Median (IQR) or N (%)
Age at start of re-RT (y)		59.8 (53.1–66.7)
Sex	Male	56 (91.8)
Smoking	Never	4 (6.6)
	Former (quit >6 months before re-RT)	21 (34.4)
	Current	24 (39.3)
	Unknown	12 (19.7)
Charlson comorbidity index	1	18 (29.5)
	2	18 (29.5)
	≥3	25 (41.0)
LRR/SP HNC site	Oropharynx	18 (29.5)
	Tongue/floor of mouth	16 (26.2)
	Neck only	10 (16.4)
	Larynx	8 (13.1)
	Hypopharynx	3 (4.9)
	Nasopharynx	3 (4.9)
	Other	3 (4.9)
Recurrent histology	SCC	62 (100.0)
Presentation type	LRR	40 (65.6)
	SP	21 (34.4)
rT stage	T0	10 (16.4)
	T1	3 (4.9)
	T2	10 (16.4)
	T3	7 (11.5)
	T4	31 (50.8)
rN stage	N0	33 (54.1)
	N1	4 (6.6)
	N2	22 (36.1)
	N3	2 (3.3)
MIRI RPA classification	Class I	13 (21.3)
	Class II	35 (57.4)
	Class III	13 (21.3)
Disease-free interval	≤24 months	35 (57.4)
Time from the end of initial RT to the diagnosis of first LRR HNC (months)		10.5 (4.8–20.4)
Time from the end of initial RT to the diagnosis of first SP HNC (months)		93.6 (52.8–127.6)

IQR, interquartile range; RT, radiotherapy; re-RT, re-irradiation; MIRI RPA, recursive partitioning analysis classification in analogy to the definition by the Multi-Institution Reirradiation, LRR, locoregional recurrence; SP, second primary; HNC, head and neck cancer; SCC, squamous cell carcinoma.

### 3.2 Treatments characteristics

All patients completed the planned first course of definitive (19.7%) or postoperative (80.3%) radio(chemo) therapy to a median dose of 64.0 Gy (IQR, 56.0–68.0) administered using conventional single fractionation (**Table 2** and **Supplementary Table 1**). Eighteen patients (29.5%) received chemotherapy with their initial course of radiotherapy (**Table 2** and **Supplementary Table 1**). Further characteristics of the first treatment are detailed in **Supplementary Table 1**.

**Table 2:** Treatment characteristics (n=61).

		Median (IQR) or N (%)	
First treatment	RT alone	3 (4.9)
	Surgery + RT	40 (65.6)
	Chemotherapy + RT	9 (14.8)
	Surgery + chemotherapy + RT	9 (14.8)
	RT dose (Gy)	64.0 (56.0–68.0)
Re-treatment	Re-RT alone	4 (6.6)
	Surgery + re-RT	13 (21.3)
	Systemic therapy + re-RT	27 (44.3)
	Surgery + systemic therapy + re-RT	17 (27.9)
Surgery	Both primary site + lymph node	5 (16.7)*
	Only primary site	17 (56.7)*
	Only lymph node	8 (26.7)*
	R0	5 (16.7)*
	R1	14 (46.7)*
	R2	11 (36.7)*
	Extracapsular extension	8 (61.5)^†^
Chemotherapy	No indication	4 (6.6)
	Indicated but not administered	16 (26.2)
	Age	7
	Comorbidity	4
	Patient refusal	3
	Weight loss	1
	Tumor progression	1
	Early terminated	17 (27.9)
	Toxicity of chemotherapy	11
	Deterioration of general condition	2
	Tumor progression	2
	Comorbidity	1
	Weight loss	1
	Administered as planned	24 (39.3)
Systemic therapy agents	Cis-5FU	13 (29.5)^‡^
	Carbo-5FU	14 (31.8)^‡^
	From Cis-5FU switch to Carbo-5FU	12 (27.3)^‡^
	Cis	2 (4.5)^‡^
	Cetuximab	3 (6.8)^‡^
Time interval between RT courses (months)		23.6 (8.4–76.6)
Re-RT dose (Gy)		60 (54.6–60.0)
Re-RT dose (Gy)	=60	42 (68.9)
	≥50 and <60	10 (16.4)
	<50, early terminated due to:	9 (14.8)
	Tumor progression	4
	Comorbidity	2
	Toxicity of chemotherapy	2
	Weight loss	1
Cumulative lifetime dose (Gy)		120.0 (116.0–126.0)
Overlap ≥50 Gy isodose (cm^3^)^§^		177.4 (109.7–262.2)
Overlap ≥60 Gy isodose (cm^3^)^¦^		39.8 (23.8–79.5)

IQR, interquartile range; RT, radiotherapy; re-RT, re-irradiation; Cis-5FU, cisplatin plus fluorouracil; Carbo-5FU, carboplatin plus fluorouracil; Cis, cisplatin. * Percentage of postoperative patients (N=30). † Percentage of patients who underwent neck surgery (N=13). ‡ Percentage of those receiving systemic therapy (N=44). § Referred to patients with electronically available dose plans and who received a complete course of re-RT therapy (N=35). ¦ Referred to 23 patients in which an overlapping volume for the ≥ 60 Gy isodose was observed.

**Table 2** reports details of the re-treatment and **Supplementary Table 6** shows distribution of patient, tumor and treatment characteristics between subgroups of patients divided according to re-RT dose. The median time interval between radiation courses was 23.6 months (IQR, 8.4–76.6). Only 52 patients (85.2%) completed all planned fractions for re-RT, 42 of whom (68.9%) were re-irradiated with a dose of 60 Gy and 10 patients (16.4%) with a lower dose (50.4–59.0 Gy) because of concerns for organs at risk. In nine of these ten patients a Charlson comorbidity index of ≥3 was observed. In nine patients (14.8%), re-RT was stopped prematurely after receiving a median dose of only 30.0 Gy (IQR, 19.8–38.9) due to tumor progression (n=4), comorbidity (n=2), toxicity of chemotherapy (n=2) and weight loss (n=1). Except for one of these nine patients, the tumor exhibited T4 stage. The median cumulative absolute dose of initial RT and re-RT was 120.0 Gy (IQR, 116.0–126.0). In 35 of 52 patients (67.3%) with electronically available dose plans and who received a complete course of re-RT, the median overlapping volume for ≥50 Gy isodose was 177.4 cm^3^ (IQR, 109.7–262.2) and 28.4% (IQR, 16.4%–39.5%) of the PTV of the first RT was covered by the ≥50 Gy isodose of the second treatment. In 23 patients (44.2%), an overlapping volume for the ≥60 Gy isodose was observed. As a result, 39.8 cm^3^ (IQR, 23.8–79.5) and 7.5% (IQR, 2.7%–14.2%) of the PTV of the initial RT were covered by the ≥60 Gy isodose of the re-RT plan. Three patients received elective irradiation of the first uninvolved nodal level. In these patients, a LRR HNC was not observed but a SP HNC was found with a median time interval of 122.9 months between the radiation courses.

In 30 patients (49.2%), surgery was performed immediately before re-RT and resulted in positive margins (R1–2) in 25 out of 30 cases (83.3%). All but one of the five patients with negative margins died due to tumor-unrelated causes and three presented SP HNC. Characteristics of the R0-resected patients are detailed in **Supplementary Table 7**. Among 28 patients with lymph node involvement, eleven patients (39.3%) underwent functional neck dissection and two patients (7.1%) diagnostic lymph node extirpation. Eight of thirteen patients (61.5%) who underwent neck surgery had an extracapsular extension and seven of these patients also had positive resection margins. Thirteen of the 30 patients who underwent surgery (43.3%) had T4 tumor (vs. 58.1% in the patients who received definitive treatment; **Supplementary Table 8**). The majority of the resected tumors (66.7%) was localized in the oropharynx, tongue and floor of the mouth. The five patients with a Charlson comorbidity index of ≥5 and the three patients with ≥80 years at re-RT start underwent surgery. **Supplementary Table 8** summarizes the distribution of patient, tumor and treatment characteristics between subgroups of patients divided according to the performance of surgery and the resection margin status.

Forty-one patients (67.2%) received concurrent chemotherapy, seven of whom received only one of the two planned cycles due to hematologic toxicity (n=4), comorbidities (n=1), weight loss (n=1) and tumor progression (n=1). Within the group of 20 patients who did not receive chemotherapy, one patient had no indication, three patients had received Cetuximab and in 16 patients concurrent chemotherapy was indicated, but not applied due to older age (n=7), comorbidity (n=4), patient refusal (n=3), weight loss (n=1), and tumor progression (n=1). After ending concurrent chemotherapy, four patients were not able to continue with adjuvant chemotherapy due to hematologic toxicity (n=3) and a deteriorating general condition (n=1). Adjuvant chemotherapy was given to 26 patients. Six of those patients were not able to complete the prescribed treatment, four because of hematologic toxicity, one because of general condition and one because of tumor progression. Twelve patients (27.3%) who started chemotherapy with Cis-5FU had to switch to Carbo-5FU because of renal failure. Distribution of patient, tumor and treatment characteristics between subgroups of patients divided according to the administration of chemotherapy is reported in **Supplementary Table 9**. Between these three groups, a significantly difference was observed in the distribution of the Charlson comorbidity index. Patients in whom chemotherapy was indicated but not administered presented a higher percentage of Charlson comorbidity index ≥3 (62.5%) compared to those in whom chemotherapy was early terminated (29.4%) and to those in whom chemotherapy was administered as planned (37.5%). Moreover, the distribution of performance of surgery was unbalanced (p=0.011). Thirteen of the 16 patients in whom chemotherapy administration was not possible (81.1%), had undergone surgery (vs. 39% of the patients who received partial or complete chemotherapy underwent surgery). In these 13 patients, the chemotherapy administration was not possible due to advanced age (n=6), comorbidity (n=4), patient refusal (n=2) and tumor progression (n=1).

### 3.3 Clinical outcomes

**Figure 1:**
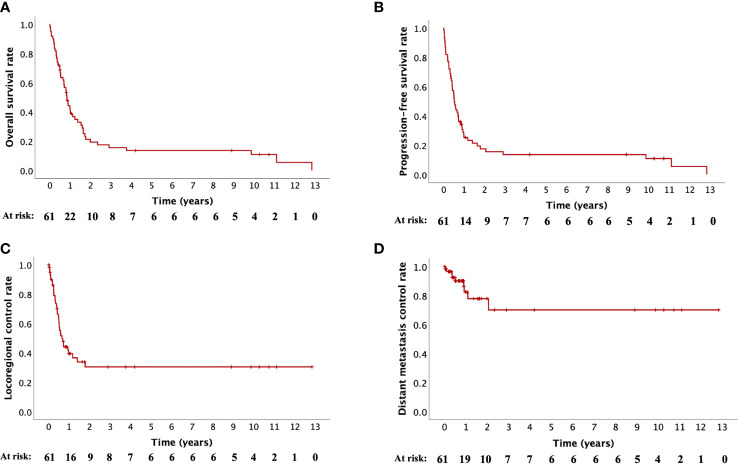
Kaplan-Meier curves for **(A)** overall survival, **(B)** progression-free survival, **(C)** locoregional control and **(D)** distant metastasis control.

The median follow-up from the re-RT start was 9.8 months (IQR, 4.4–19.4). Six patients (9.8%) were followed up for more than nine years and four patients (6.6%) for more than ten years. All patients who survived more than nine years presented neither locoregional nor distant progression at the last follow-up. Further characteristics regarding these patients are reported in **Supplementary Table 10**. Fifty-two patients (85.2%) died due to tumor progression of cancer treated with re-RT and its complications (n=36), hematologic toxicity (n=1) or other causes (n=15). The median time to death from the re-RT start was 9.0 months (IQR, 3.9–17.9) and in 19 patients it was less than six months. The median OS was 10.1 months (IQR, 4.4–20.7), with 1-, 2- and 5-year survival rates of 40.6%, 19.4% and 13.6%, respectively (**Figure 1A** and **Supplementary Figure 1A**).

Forty patients (65.6%) developed tumor progression. The progression-free rates at 1, 2 and 5 years were 25.3%, 17.5% and 13.6%, respectively and the median PFS was 6.3 months (IQR, 3.0–14.1; **Figure 1B**, **Supplementary Figure 1B**). Locoregional failure was the most common, occurring in 36 patients (59.0%) and at a median of 5.1 months (IQR, 3.0–7.7). Distant failure was observed in nine patients (14.8%) at a median of 6.0 months (IQR, 2.7–12.0). Five patients (8.2%) developed both locoregional and distant failure. Distant metastases were diagnosed in the lungs (n=3), bones (n=2), axillary lymph nodes (n=2), liver (n=1) and simultaneously in the lungs, mediastinal lymph nodes as well as liver (n=1). The median LRC was 7.9 months (IQR, 4.1–(>154)), with 1-, 2- and 5-year LRC rates of 39.5%, 30.4% and 30.4%, respectively (**Figure 1C** and **Supplementary Figure 1C**). Statistical analysis of DMC was not performed due to the small number of events (n=9) (**Figure 1D** and **Supplementary Figure 1D**).

**Figure 2:**
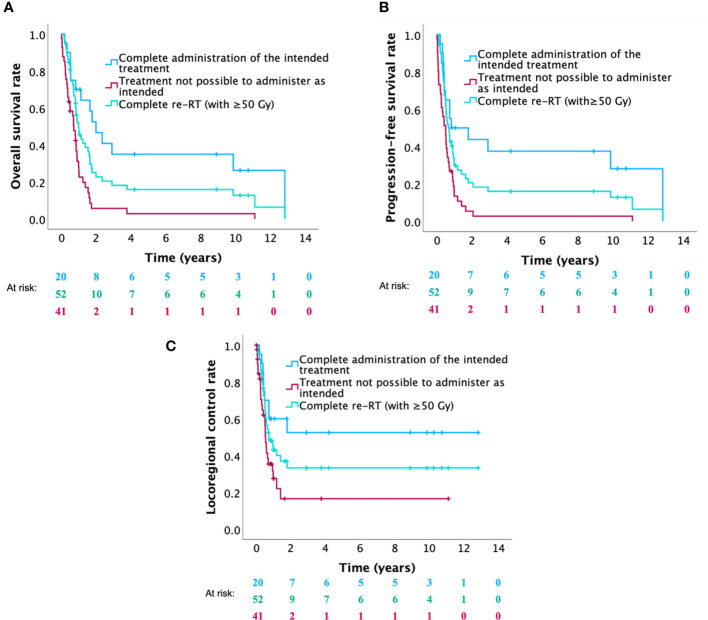
Kaplan-Meier curves for **(A)** overall survival, **(B)** progression-free survival and **(C)** locoregional control of patients who received the complete intended treatment (re-RT with 60 Gy combined with chemotherapy) versus patients who were not able to receive the complete intended treatment (no or incomplete chemotherapy and/or re-RT with <60 Gy) versus patients who completed re-RT (with ≥50 Gy).

**Figure 3:**
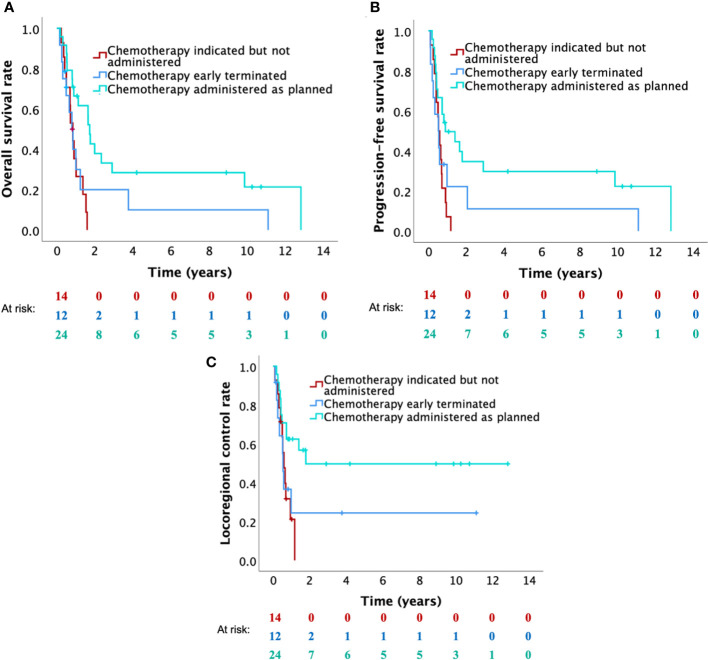
Kaplan-Meier curves for **(A)** overall survival, **(B)** progression-free survival and **(C)** locoregional control of patients who completed re-RT (with 50 Gy), further subdivided according to the administration of chemotherapy (patients for whom chemotherapy was indicated but not possible versus patients who early terminated chemotherapy versus patients who were able to complete chemotherapy).

Clinical outcomes of different groups of patients are summarized in **Table 3** (for OS) and **Supplementary Tables 11, 12** (for PFS and LRC). In the 20 patients (32.8%) receiving the complete planned treatment (hyperfractionated re-RT with 60 Gy plus concurrent and adjuvant platinum-based chemotherapy), 2-year OS, PFS and LRC rates were 46.7%, 43.8%, and 52.5%, respectively (**Figures 2A–C**). In patients in whom it was not possible to administer the complete planned treatment (n=41, 65.6%), 2-year OS, PFS and LRC rates of 5.7%, 5.4% and 16.5%, were observed, respectively (**Figures 2A–C**). Patients who completed re-RT (with ≥50 Gy; n=52) showed 1- and 2-year OS, PFS and LRC rates of 47.6% and 22.7%, 29.7% and 20.6%, and 43.1% and 33.3%, respectively (**Figures 2A–C**). Among these patients, 24 patients (46.2%) received chemotherapy as planned with 1- and 2-year OS, PFS and LRC rates of 66.4% and 37.9%, 49.7% and 34.8%, and 62.5% and 49.7%, respectively (**Figures 3A–C**). In patients in whom chemotherapy was not possible (n=14, 26.9%), clinical outcomes were significantly lower, presenting 1- year OS, PFS and LRC rates of 35.4%, 7.1% and 21.2% (**Figures 3A–C**). Kaplan-Meier curves for OS, PFS and LRC of the patients who received re-RT with 60 Gy as intended, further subdivided according to the administration of chemotherapy, are shown in **Supplementary Figures 2A–C**. In patients who completed re-RT (with ≥50 Gy) and who underwent surgery (n=26, 50.0%), OS rates were significantly not better than those observed in the group of patients receiving definitive (chemo)-re-RT (n=26, 50.0%) with 1- and 2-year OS rates of 47.8% and 14.5% versus 47.4% and 30.2%, respectively. In the nine patients who underwent surgery, received partial or complete chemotherapy and completed re-RT with 60 Gy, 1-, 2- and 5-year OS rates were 76.2%, 45.7% and 45.7%, respectively.

**Table 3:** Overall survival (OS) outcomes of the entire cohort (A) and of different groups of patients divided according to the completion of treatment (completion of re-RT with ≥50 Gy (B), with 60 Gy (C) and with 60 Gy plus partial or complete chemotherapy (D)). Further divisions of each group regarding chemotherapy, surgery and MIRI RPA classification were performed.

	1-, 2-, 5-year OS%	OS in months median (IQR)	HR (95% CI)	P			
A) All patients (N=61)	40.6, 19.4, 13.6	10.1 (4.4–20.7)					
**Completion of the intended treatment (No vs. Yes)**			3.402 (1.752–6.605)	<.001
No (n=41)	25.5, 5.7, 0.0	8.5 (3.7–12.2)		
Yes (n=20)*	70.0, 46.7, 35.0	23.8 (6.4–154.1)	Ref.			
**Chemotherapy**				
No, although indicated (n=16)	30.9, 0.0, 0.0	8.5 (4.4–12.2)	Ref.	.002
Early terminated (n=17)	21.2, 14.1, 7.1	5.9 (2.5–12.0)	0.93 (0.44–1.97)	.857
Yes, as planned (n=24)	66.4, 37.9, 28.5	20.7 (9.7–118.5)	0.32 (0.15–0.68)	.003
**Re-RT completion (<50 vs. ≥50 Gy)**			20.12 (7.28–56.4)	<.001
No, re-RT dose <50 Gy (n=9)	0.0, 0.0, 0.0	0.9 (0.5–2.7)		
Yes (n=52)	47.6, 22.7, 15.9	11.8 (6.4–21.1)	Ref.	
≥50 and <60 Gy (n=10)	30.0, 0.0, 0.0	9.7 (7.8–12.1)		
60 Gy (n=42)	52.3, 29.0, 20.3	13.5 (6.4–34.8)		
**Surgery (Yes vs. No)**			1.16 (0.67–2.01)	.600
No (n=31)	39.7, 25.3, 14.5	10.8 (4.0–28.1)	Ref.	
Yes (n=30)	41.5, 12.6, 12.6	10.1 (4.4–19.7)		
**MIRI RPA classification**				
Class I (n=13)	53.8, 7.7, 7.7	12.2 (8.5–19.1)	Ref.	.137
Class II (n=35)	43.7, 30.3, 20.2	11.8 (4.3–34.8)	0.822 (0.412–1.638)	.577
Class III (n=13)	17.9, 0.0, 0.0	6.4 (2.7–10.8)	1.72 (0.75–3.94)	.201
B) Patients who completed re-RT with ≥50 Gy (N=52)	47.6, 22.7, 15.9	11.8 (6.4–21.1)					
**Chemotherapy**				
No, although indicated (n=14)	35.4, 0.0, 0.0	10.1 (6.0–16.5)	Ref.	.012
Early terminated (n=12)	30.0, 20.0, 10.0	9.5 (3.7–14.8)	0.69 (0.29–1.64)	.400
Yes, as planned (n=24)	66.4, 37.9, 28.5	20.7 (9.7–118.5)	0.31 (0.14–0.70)	.005
**Surgery (Yes vs. No)**			1.24 (0.67–2.28)	.490
No (n=26)	47.4, 30.2, 17.2	12.0 (8.4–34.8)	Ref.	
Yes (n=26)	47.8, 14.5, 14.5	10.7 (6.0–19.7)		
**MIRI RPA classification**				
Class I (n=12)	58.3, 8.3, 8.3	12.2 (8.5–19.1)	Ref.	.076
Class II (n=29)	52.7, 36.5, 24.3	14.8 (8.3–45.2)	0.69 (0.33–1.46)	.331
Class III (n=11)	21.2, 0.0, 0.0	9.5 (3.7–10.8)	1.77 (0.72–4.31)	.212
C) Patients who completed re-RT with 60 Gy as in protocol (N=42)	52.3, 29.0, 20.3	13.5 (6.4–34.8)					
**Chemotherapy**				
No, although indicated (n=11)	37.4, 0.0, 0.0	8.3 (5.0–16.5)	Ref.	.024
Early terminated (n=9)	40.0, 26.7, 13.3	12.0 (9.5–45.2)	0.52 (0.19–1.42)	.200
Yes, as planned (n=20)	70.0, 46.7, 35.0	23.8 (6.4–154.1)	0.274 (0.108–0.699)	.007
**Surgery (Yes vs. No)**			1.06 (0.52–2.16)	.866
No (n=24)	51.8, 33.0, 18.8	14.8 (6.4–34.8)	Ref.	
Yes (n=18)	53.0, 22.7, 22.7	13.5 (6.0–23.8)		
**MIRI RPA classification**				
Class I (n=8)	50.0, 12.5, 12.5	10.2 (6.0–16.5)	Ref.	.305
Class II (n=27)	56.9, 39.4, 26.2	18.4 (8.3–118.5)	0.67 (0.28–1.61)	.367
Class III (n=7)	35.7, 0.0, 0.0	9.5 (5.0–23.8)	1.40 (0.44–4.48)	.567
D) Patients who completed re-RT with 60 Gy and who received partial or complete chemotherapy (N=29)	61.3, 40.9, 28.6	19.7 (9.5–118.5)					
**Surgery (Yes vs. No)**			0.59 (0.21–1.61)	.300
No (n=20)	54.5, 38.2, 21.8	14.8 (6.3–45.2)	Ref.	
Yes (n=9)	76.2, 45.7, 45.7	23.8 (13.5–133.4)		

OS, overall survival; re-RT, re-irradiation; MIRI RPA, recursive partitioning analysis classification in analogy to the definition by the Multi-Institution Reirradiation. *Patients who received chemotherapy as planned and definitive or adjuvant re-RT with 60 Gy.

Regarding the RPA classification in analogy to the definition by the Multi-Institution Reirradiation (MIRI) Collaborative (8), the 1-year OS rates were 53.8% in class I, 43.7% in class II and 17.9% in class III. The 1-year PFS rates were 30.8% in class I, 27.2% in class II and 15.4% in class III. The 1-year LRC rates were 47.6% in class I, 38.7% in class II and 32.4% in class III.

Clinical outcomes of LRR HNC patients versus SP HNC patients are shown in **Supplementary Figure 3** and in **Supplementary Tables 13–15**. The median OS, PFS and LRC of LRR HNC patients versus SP HNC patient were 7.8 months (IQR, 3.7–20.7) versus 13.5 months (IQR, 9.7–19.7), 5.1 months (IQR, 2.3–11.8) versus 8.5 months (IQR, 4.3–14.1), and 6.2 months (IQR, 3.8–(>154)) versus 11.3 months (6.3–(>128)), respectively. The clinical outcomes between the two patient groups did not significantly differ (**Supplementary Figure 3**). Further divisions of each group regarding treatment completion, chemotherapy, surgery and MIRI RPA classification were performed and outcomes were reported in **Supplementary Tables 13–15**.

### 3.4 Univariate and multivariate analysis

Regarding OS, older age, chemotherapy administration and re-RT dose of 60 Gy (vs. <60 Gy) were independently and significantly associated with overall survival in multivariate analysis (**Table 4** and **Supplementary Figures 4A–C, 5A–C**). As for the PFS, N0–1 stage, older age, chemotherapy administration and re-RT dose of 60 Gy (vs. <60 Gy) were significantly and independently predictive for improved PFS (**Table 4** and **Supplementary Figures 4A–C, 5A–C**). Regarding LRC, N0–1 stage, older age and chemotherapy administration were independent prognostic factors for favorable LRC (**Table 4** and **Supplementary Figures 4A–C, 5A–C**). The results of the univariate analysis are shown in **Table 5**. The relationships between the different factors are presented in the **Supplementary Tables 3–6, 8, 9, 16, 17**).

**Table 4:** Multivariate analysis for overall survival, progression-free survival and locoregional control.

Variable	OS		PFS		LRC	
	HR (95% CI)	p	HR (95% CI)	p	HR (95% CI)	p
rT stage (4 vs. 0-3)	1.800 (0.825–3.926)	.140	1.563 (0.721–3.390)	.258	2.111 (0.792–5.629)	.135
rN stage (2-3 vs. 0-1)	1.644 (0.812–3.328)	.167	2.290 (1.134–4.627)	.021	4.204 (1.708–10.351)	.002
Disease-free interval (>24 vs. ≤24 months)	0.752 (0.398–1.422)	.381	0.747 (0.396–1.411)	.369	0.473 (0.213–1.050)	.066
Age (>60 vs. ≤60 years)	0.467 (0.230–0.946)	.035	0.383 (0.187–0.782)	.008	0.230 (0.093–0.572)	.002
Charlson comorbidity (≥3 vs. 1–2)	0.786 (0.328–1.882)	.588	0.645 (0.283–1.470)	.297	1.047 (0.410–2.674)	.923
Baseline dysphagia (Grade 3-4 vs. 0-2)	1.791 (0.862–3.721)	.118	1.265 (0.619–2.584)	.519	0.866 (0.359–2.089)	.749
Surgery (Yes vs. no)	0.818 (0.421–1.590)	.553	0.565 (0.283–1.129)	.106	0.548 (0.225–1.330)	.183
Chemotherapy						
Ind. but not adm.	1.000	.005	1.000	.002	1.000	<.001
Early terminated	0.670 (0.250–1.796)	.426	0.431 (0.167–1.110)	.081	0.279 (0.089–0.876)	.029
Adm. as planned	0.264 (0.104–0.672)	.005	0.209 (0.087–0.502)	<.001	0.121 (0.041–0.362)	<.001
Re-RT dose (<60 vs. 60 Gy)	3.770 (1.658–8.571)	.002	3.303 (1.516–7.199)	.003	1.373 (0.534–3.528)	.510

OS, overall survival; PFS, progression-free survival; LRC, locoregional control; MVA, multivariate analysis; HR, hazards ratio; CI, confidence interval; SP, second primary; LRR, locoregional recurrence; Ind., indicated; Adm., administered; re-RT, re-irradiation.

**Table 5:** Univariate analysis for overall survival, progression-free survival and locoregional control.

Variable	OS		PFS		LRC	
	HR (95% CI)	p	HR (95% CI)	p	HR (95% CI)	p
rT stage (4 vs. 0–3)	2.044 (1.157–3.612)	.014	2.164 (1.234–3.795)	.007	2.211 (1.134–4.312)	.020
rN stage (2–3 vs. 0–1)	1.192 (0.672–2.114)	.549	1.378 (0.786–2.415)	.263	1.645 (0.846–3.201)	.143
Disease-free interval (>24 vs. ≤24 months)	0.572 (0.325–1.008)	.053	0.630 (0.360–1.100)	.104	0.510 (0.256–1.016)	.056
Age (>60 vs. ≤60 years)	0.607 (0.345–1.068)	.084	0.541 (0.310–0.945)	.031	0.365 (0.181–0.733)	.005
Charlson comorbidity (≥3 vs. 1–2)	0.950 (0.539–1.674)	.859	0.884 (0.509–1.538)	.663	0.974 (0.502–1.888)	.938
Baseline dysphagia (Grade 3–4 vs. 0–2)	2.399 (1.280–4.495)	.006	1.763 (0.973–3.194)	.061	1.507 (0.743–3.054)	.255
Surgery (Yes vs. no)	1.159 (0.667–2.012)	.600	0.956 (0.556–1.642)	.869	0.904 (0.469–1.742)	.763
Chemotherapy						
Ind. but not adm.	1.000	.002	1.000	.004	1.000	.022
Early terminated	0.934 (0.442–1.974)	.857	0.927 (0.453–1.898)	.836	0.911 (0.389–2.133)	.831
Adm. as planned	0.317 (0.148–0.680)	.003	0.339 (0.165–0.700)	.003	0.336 (0.144–0.785)	.012
Re-RT dose (60 vs. <60 Gy)	0.296 (0.161–0.543)	<.001	0.369 (0.206–0.661)	<.001	0.609 (0.291–1.272)	.187

OS, overall survival; PFS, progression-free survival; LRC, locoregional control; UVA, univariate analysis; HR, hazards ratio; CI, confidence interval; SP, second primary; LRR, locoregional recurrence; Ind., indicated; Adm., administered; re-RT, re-irradiation.

### 3.5 Predictors of therapy completion

Outcomes of univariate and multivariable logistic regression analysis of predictors of treatment completion (re-RT dose of 60 Gy plus early terminated or completed as planned chemotherapy) are reported in **Tables 6**, **7**, respectively. The multivariable logistic regression model showed that the odds of treatment completion in patients with Charlson comorbidity index of 1–2 was 5.08 (1/0.197) times higher than that in patients with Charlson comorbidity index ≥3 (odd ratio [OR], 0.197; 95% CI, 0.051–0.757; p=0.018). Patients who did not undergo surgery had odds 4.18 times higher compared to the postoperative patients in terms of treatment completion (odd ratio, 0.239; 95% CI, 0.074–0.769; p=0.016).

**Table 6:** Univariate logistic regression analysis with treatment completion (re-RT dose of 60 Gy plus early terminated or completed as planned chemotherapy) as dependent variable and one possible predictor as independent variable.

	Treatment completion		OR (95% CI)	P
	Yes (N=29) n (%)*	No (N=32) n (%)*		
rT stage (4 vs. 0–3)			1.395 (0.509–3.825)	.518
T0 (n=10)	5 (17.2)	5 (15.6)		
T1 (n=3)	1 (3.4)	2 (6.3)		
T2 (n=10)	3 (10.3)	7 (21.9)		
T3 (n=7)	4 (13.8)	3 (9.4)		
T4 (n=31)	16 (55.2)	15 (46.9)		
rN stage (2–3 vs. 0–1)			0.677 (0.240–1.908)	.460
N0 (n=33)	17 (58.6)	16 (50.0)		
N1 (n=4)	2 (6.9)	2 (6.3)		
N2 (n=22)	9 (31.0)	13 (40.6)		
N3 (n=2)	1 (3.4)	1 (3.1)		
Disease-free interval (mo) (>24 vs. ≤24)			1.187 (0.430–3.282)	.740
≤24 months (n=35)	16 (55.2)	19 (59.4)		
>24 months (n=26)	13 (44.8)	13 (40.6)		
Charlson comorbidity index (≥3 vs. 1–2)			0.247 (0.082–0.744)	.013
1–2 (n=36)	22 (75.9)	14 (43.8)		
≥3 (n=25)	7 (24.1)	18 (56.3)		
Age at start of re-RT (y) (>60 vs. ≤60)			0.813 (0.297–2.226)	.686
≤60 (n=32)	16 (55.2)	16 (50.0)		
>60 (n=29)	13 (44.8)	16 (50.0)		
Baseline dysphagia (3–4 vs. 0–2)			0.727 (0.244–2.170)	.568
Grade 0–2 (n=42)	21 (72.4)	21 (65.6)		
Grade 3–4 (n=19)	8 (27.6)	11 (34.4)		
Surgery (Yes vs. No)			0.236 (0.081–0.689)	.008
No (n=31)	20 (69.0)	11 (34.4)		
Yes (n=30)	9 (31.0)	21 (65.6)		

OR, odds ratio, which were calculated using the logistic regression; CI, confidence interval. * Percentage refers to completion of therapy.

**Table 7:** Multivariable logistic regression analysis with treatment completion (re-RT dose of 60 Gy plus early terminated or completed as planned chemotherapy) as dependent variable and multiple possible predictors as independent variables.

	Treatment completion		OR (95% CI)	P
	Yes (N=29) n (%)*	No (N=32) n (%)*		
rT stage (4 vs. 0–3)			.670 (0.191–2.355)	.532
T0 (n=10)	5 (17.2)	5 (15.6)		
T1 (n=3)	1 (3.4)	2 (6.3)		
T2 (n=10)	3 (10.3)	7 (21.9)		
T3 (n=7)	4 (13.8)	3 (9.4)		
T4 (n=31)	16 (55.2)	15 (46.9)		
rN stage (2–3 vs. 0–1)			0.833 (0.254–2.732)	.763
N0 (n=33)	17 (58.6)	16 (50.0)		
N1 (n=4)	2 (6.9)	2 (6.3)		
N2 (n=22)	9 (31.0)	13 (40.6)		
N3 (n=2)	1 (3.4)	1 (3.1)		
Disease-free interval (mo) (>24 vs. ≤24)			1.365 (0.394–4.725)	.624
≤24 months (n=35)	16 (55.2)	19 (59.4)		
>24 months (n=26)	13 (44.8)	13 (40.6)		
Charlson comorbidity index (≥3 vs. 1–2)			0.197 (0.051–0.757)	.018
1–2 (n=36)	22 (75.9)	14 (43.8)		
≥3 (n=25)	7 (24.1)	18 (56.3)		
Age at start of re-RT (y) (>60 vs. ≤60)			1.052 (0.312–3.544)	.935
≤60 years (n=32)	16 (55.2)	16 (50.0)		
>60 years (n=29)	13 (44.8)	16 (50.0)		
Baseline dysphagia (3–4 vs. 0–2)			0.825 (0.217–3.134)	.778
Grade 0–2 (n=42)	21 (72.4)	21 (65.6)		
Grade 3–4 (n=19)	8 (27.6)	11 (34.4)		
Surgery (No vs. Yes)			0.239 (0.074–0.769)	.016
No (n=31)	20 (69.0)	11 (34.4)		
Yes (n=30)	9 (31.0)	21 (65.6)		

OR, odds ratio, which were calculated using the logistic regression; CI, confidence interval. * Percentage refers to completion of therapy.

### 3.6 Toxicity

Patients who terminated the re-RT early (n=9) were excluded from the evaluation of toxicity. Fifty-four patients (88.5%) and 41 patients (67.2%) were evaluable for acute and late toxicity, respectively. No re-RT-related acute and late grade 5 toxicities were observed. Before re-RT, 17 patients (31.5%) exhibited grade 3–4 dysphagia (**Supplementary Table 18**). Subtracting baseline toxicities, the most common new grade ≥3 acute toxicities were dysphagia and mucositis affecting 27 (51.9%) and 24 patients (46.2%), respectively (**Table 8**). In two of the 41 evaluable patients (4.9%), re-RT-related grade 3–4 dysphagia persisted until the last follow-up (**Table 8**). More detailed information about the changes in grades of dysphagia according to baseline dysphagia is reported in **Table 9**. Four patients (9.8%) developed osteoradionecrosis at a median of 9.7 months (IQR, 4.4–76.3) after receiving a median cumulative absolute dose of 116.0 Gy (IQR, 114.8 Gy–119.0 Gy) and required surgery. In the six patients (9.8%) who survived more than nine years, no late toxicity grade ≥3 was observed, except for osteoradionecrosis in two patients (**Supplementary Table 10**). **Supplementary Table 18** illustrates the distribution of baseline, acute and late toxicities grades without the subtraction of toxicity already present before re-RT.

**Table 8:** Acute and late re-RT-related toxicities, subtracting baseline toxicity.

	New grade	
	**1-2**	**3-4**
Acute toxicity (n=52) (N, %)		
Dysphagia	5 (9.6)	27 (51.9)
Xerostomia	20 (38.5)	1 (1.9)
Dysgeusia	25 (48.1)	–
Radiation dermatitis	31 (59.6)	2 (3.8)
Mucositis	15 (28.8)	24 (46.2)
Fibrosis	1 (1.9)	–
Telangiectasia	2 (3.8)	–
Late toxicity (n=41) (N, %)*		
Osteoradionecrosis	–	4 (9.8)
Dysphagia	7 (17.1)	2 (4.9)
Xerostomia	11 (26.8)	–
Dysgeusia	13 (31.7)	–
Fibrosis	14 (34.1)	–
Telangiectasia	8 (19.5)	–

* For nine of 41 evaluable patients data were unknown.

**Table 9:** Acute and late dysphagia according to baseline dysfunction.

	Baseline dysphagia*			
	Grade 0–2	Grade 3	Grade 4	
Acute dysphagia (n=52) (n, %)				Total
Grade 0-2	8	–	–	8
Grade 3	19	8	–	27
Grade 4	8	6	1	15
Unknown	–	1	1	2
Total	35	15	2	52
Late dysphagia (n=41) (n, %)^†^				
Grade 0-2	22	3	–	25
Grade 3	1	1	–	2
Grade 4	1	4	–	5
Unknown	5	3	1	9
Total	28	11	1	41

*Excluded patients who terminated prematurely re-RT. ^†^For nine of 41 evaluable patients data were unknown.

Toxicity distribution in LRR HNC patients versus that in SP HNC patients is summarized in **Supplementary Tables 19, 20**. Although statistical comparisons between the two groups of patients are difficult due to the limited number of evaluable patients, similar outcomes were observed. However, slight differences were observed in acute dysphagia grade 3–4 and acute mucositis grade 3–4, developed in 66.7% and 55.6% of SP HNC patients versus 44.1% and 35% of LRR HNC patients, respectively.

Among the patients who received chemotherapy (n=41, 67.2%), twelve patients (29.3%) had hematologic toxicities requiring treatment discontinuation (in nine patients chemotherapy was interrupted early and in two patients both chemotherapy and re-RT) and in one case leading to death.

Median weight change (from pre-treatment baseline) was -2.1% (IQR, -5.4% to +1.6%) in 32 patients with available data.

## 4 Discussion

In the present analysis reporting our experience with LRR/SP HNC patients in whom the treatment protocol and techniques remained the same throughout the years (hyperfractionated re-RT with a total dose of 60 Gy combined with platinum-based chemotherapy), the 2-year OS, LRC and PFS rates were 19%, 30% and 18%, respectively. Considering the entire cohort without looking at differences between studies, these outcomes are slightly lower than those typically reported in other studies based on IMRT with a 2-year OS rate of 29%–75.7% (16, 18, 19, 24, 25, 27, 36), 2-year LRC rate of 35.9%–65% (18, 20, 39) and 2-year PFS rate of 20%–32% (21, 23, 35, 39). However, the 2-year OS rate observed in our analysis is more favorable than that found in the Radiation Therapy Oncology Group (RTOG) 9610, which reported a 2-year OS of 15% for patients treated with hyperfractionated re-RT using a three-dimensional conformal technique (10). The 2-year PFS rate in this analysis is very similar to the one found in a recent large retrospective and multi-institution analysis including 253 patients who underwent definitive radio(chemo)therapy, reporting a 2-year PFS rate of 19% (27). However, looking at the patients at risk after five and ten years, our results were encouraging. In the mentioned study including 253 patients, the patients at risk for overall survival after five years were three (1%; vs. six patients (10%) in our study) (27). In the present analysis, most of the patients alive three years after the start of re-RT, were still alive and disease-free with acceptable toxicity after nine years, showing that hyperfractionated re-RT is a feasible option for carefully selected patients. Moreover, the patients (n=20, 33%) in whom the administration of the complete treatment (re-RT with 60 Gy plus chemotherapy completed as planned) was possible, showed significantly better outcomes compared to those in whom it was not possible (2-year OS rate of 46.7% versus 5.7%). Therefore, the present analysis highlights the importance to select as best as possible the patients who will be able to complete the therapy. There are also controversies on the use of adjuvant systemic therapy, and since inclusion of the last patient into this analysis, the treatment landscape of loco regionally recurrent or persistent disease has changed significantly since the advent of immunotherapy, in particular in combination with stereotactic body radiotherapy (SBRT) (9). The fact that all patients were treated in the same manner, and with adjuvant chemotherapy, may help to better understand the presented results. It also highlights that treatment intensification has its limitations in terms of improving outcome while adding additional toxicity.

Our study showed that the Charlson comorbidity index was an important predictor of treatment completion (defined as receiving re-RT with 60 Gy plus at least one cycle of chemotherapy). Patients with a higher Charlson comorbidity index had a higher probability of not being able to receive and complete the treatment as intended. This finding could partly explain why patients who were unable to receive chemotherapy (vs. patients who received chemotherapy) had significantly worse survival outcomes in both univariate and multivariate analysis. Moreover, it confirmed the finding of other studies, stating that comorbidity is an important prognostic factor for survival outcomes in patients undergoing re-RT (32, 40).

Similarly to chemotherapy, Charlson comorbidity index could explain why the ten patients who completed re-RT with a dose between 50 Gy and 60 Gy presented very poor 1- and 2-year OS rates of 30.0% and 0%, respectively. Indeed, it was found that nine of the ten patients exhibited a Charlson comorbidity index ≥3. The fact that the patients receiving <50 Gy showed worse survival outcomes was not surprising since re-RT has been discontinued, mostly due to tumor progression and comorbidity, increasing the risk of death. Therefore, comparisons between patients who received a total dose of 60 Gy versus patients who received lower doses are limited and difficult in the present study. Since the prescribed dose was 60 Gy for both postoperative and definitive re-RT as in the prospective RTOG 9610 and RTOG 9911 trials (10, 11), we could not confirm the result of a recent large multi-institution analysis in line with another report stating that in general 60 Gy are not sufficient for definitive re-RT, prescribing, therefore, doses of ≥66 Gy (20, 41). Moreover, in the mentioned study (41), hyperfractionation was not associated with improved LRF or OS and was associated with greater grade ≥3 late toxicity in postoperative patients compared to patients treated with conventionally fractionated re-RT. Conversely, in a recent study hyperfractionation (vs. standard fractionation) led to a significantly better 3-year OS rate (74.6% vs. 55%) in patients with locally advanced recurrent nasopharyngeal carcinoma, suggesting that hyperfractionated IMRT could be used as the standard of care for these patients (42). In our study, the only three patients presenting nasopharyngeal tumors died within 14 months after re-RT with 60 Gy, due to tumor progression. Further similar studies must clarify the role of hyperfractionation in the reirradiation setting of non-nasopharyngeal HNCs.

In addition to Charlson Comorbidity index, surgery was another predictor of treatment completion: patients who received definitive (chemo)-re-RT had a significantly higher probability to complete treatment compared to postoperative patients. The reason for this outcome could lie in the fact that in 43% of the postoperative patients (vs. 9.7% in the subgroup of patients receiving definitive re-RT, p=0.019) it was not possible to administer chemotherapy, due mostly to advanced age or comorbidity.

This consideration on postoperative patients should also be taken into account when considering the influence of surgery on survival outcomes in our study, together with other factors discussed below. In many studies, surgery prior to re-RT exhibited an association with improved OS, PFS and LRC (8, 18, 21, 31, 33–35). However, this association could be a result of an intrinsic bias of the retrospective nature of these studies. Indeed, surgical candidates present usually smaller tumor volumes and a higher Karnofsky Performance Status, as declared by the authors themselves. In our analysis, surgery prior to re-RT resulted in low R0 resection rate (17% of the postoperative patients) and was not associated with improved OS, PFS and LRC. The fact that only 17% of the postoperative patients showed negative resection margin could have influenced this outcome. A recent study on salvage surgery showed that patients with postoperative positive resection margins had poor survival outcomes that were not significantly improved with adjuvant (chemo)reirradiation (43). In a study on resection margins in oral cancer surgery, it was observed that advanced tumor size and stage was associated with a higher number of inadequate resection margins. In our analysis, a high presence of large tumor size (T3-4; 56%) and advanced tumor stage (stage IV; 73%) among surgical patients may have led to a high percentage of positive resection margins (44). A similar R0 resection rate (19% of the postoperative patients) was observed in another re-RT study, in which 22 of the 257 patients exhibited negative margins (33). In other studies, the R0 resection rate varies between 30%-67% of the postoperative patients and between 17%-32% of the entire cohort (8, 25, 32, 34–36). In the study of Bots et al., 31% of patients receiving postoperative re-RT presented a clear resection margin (34). In this case, the percentage of large tumor size (T3-4) was lower (42%) than those observed in our analysis (34). In Ward et al., 67% of the surgical patients had no gross residual disease at the time of re-RT (8). 45% of all patients included in the study (treated with postoperative or definitive re-RT) exhibited advanced tumor (T3-4) (8). By comparing the resection margin status of the present analysis with those of others, it should be also considered that our cohort consisted mostly of advanced tumors. In addition, the low rate of clear margin in the present analysis could be partly explained by the fact that 20% of postoperative patients had already had a previous LRR/SP HNC, that could be often R0-resected. A re-RT was waived to treat this R0-resected tumor; however, re-RT was performed to treat the following LRR/SP HNC where the chance of R0-resection was then more difficult. In this context, it should be also mentioned that, since our institution was one of the first institutions in the region to introduce IMRT (on 7.10.2002), patients with challenging clinical scenarios of other institutions were treated at this Hospital. Therefore, a high rate of R1–2 could be observed.

In multivariate analysis, older patients showed improved survival outcomes. The fact that younger age was significantly associated with incomplete chemotherapy, could explain this outcome, highlighting the importance of finding a predictor of treatment completion.

Looking at the tumor-related factors, the present analysis highlighted the importance to consider T- and N stages for decision-making, confirming the results of previous studies (19, 32, 33). In fact, the N0–1 stage (vs. N2–3) had significantly better PFS and LRC in multivariate analysis, and the T0–3 (vs. T4) stage had significantly better OS, PFS and LRC in univariate analysis. For this reason, it is pivotal, in post-treatment surveillance, to early identify LRR/SP HNC when the prognosis is still superior (45). In this regard, the fact that a T4 LRR/SP HNC was present in 28% of the patients with Charlson comorbidity index ≥3 (vs. 66.7% in patients with an index of 1–2) may have influenced the clinical outcomes of the two groups. This should be taken into account by looking to the outcomes of both the univariate and multivariate analysis in regard to the Charlson comorbidity index which was not a prognostic factor for OS, PFS and LRC. In the present analysis, we were unable to statistically investigate the association between the anatomical site of LRR/SP HNC and survival outcomes, due to the uneven distribution of tumors at a particular subsite. It is known that oral cavity and hypopharyngeal tumors exhibit relatively poor prognosis, whereas nasopharyngeal, laryngeal cancer or lateral neck recurrence have a better prognosis (29). The lower portion of cancer in the nasopharynx and larynx (18%) in this study, compared to those in other analyses ranging from 19% to 46%, could have contributed to worse survival outcomes (15, 19, 21, 23, 24, 33, 35, 36, 46). We can furthermore note that in the recent large multi-institutional study mentioned above with similar results to ours, nasopharyngeal cancers were excluded from the study (27). Moreover, all laryngeal tumors (n=8) and two of the three nasopharyngeal tumors analyzed in the present study exhibited T4 stage. A disease-free interval >24 months was significantly associated with improved OS and LRC in univariate analysis and showed a trend of improved LRC in multivariate analysis. Therefore, in line with several other reports, our study supports the disease-free interval (roughly comparable to the time interval between RT courses analyzed in other studies) as a prognostic factor, reflecting the biological aggressiveness of LRR/SP HNC (8, 19, 32, 33, 35). In the univariate analysis, a negative influence of baseline grade 3–4 dysphagia on OS and PFS was observed. Also, in the RPA defined by the Multi-Institution Reirradiation (MIRI) Collaborative, if the time interval between RT courses is ≤24 months, organ dysfunction is an important factor for OS, suggesting that it is a more useful indicator than general performance, since it refers to the tumor location and degree of invasion (20). Another study specified that organ dysfunctions may be a marker of aggressive disease biology (30). In fact, more aggressive diseases require more extensive previous treatments, which results in more relevant organ dysfunctions (30). As mentioned above, more aggressive LRR/SP HNC occurred in a time interval of ≤24 months from the previous diagnosis, and, therefore, it is not surprising that in our study 79% of patients with baseline grade 3–4 dysphagia (vs. 48% of patients with grade 0–2 dysphagia) showed a disease-free time interval of ≤24 months.

The absence of re-RT-related acute and late grade 5 toxicity in our study confirmed that re-RT with IMRT contributed to improvement in safety compared to pre-IMRT modalities (10, 11, 18). Whether hyperfractionated IMRT (vs. standard fractionation) have also contributed to this outcome, we were unfortunately unable to demonstrate. In this regard, a recent study showed that hyperfractionated IMRT could significantly reduce the incidence of late radiation-induced toxicities (42). In the present study, the most common new grade 3–4 acute toxicity was dysphagia, affecting 52% of the patients. These high rates of acute dysphagia compared to the lower ones reported in other studies (19, 25, 26, 39) may be explained by several factors. One possible reason is that the toxicity was prospectively scored, which may have led to a more sensitive and accurate detection of dysphagia than in retrospective studies. Another explanation could lie within the large proportion of oropharynx and oral cavity (tongue/floor of the mouth) cancer (56%) in our cohort, which may intrinsically have led to worse dysphagia. In this regard, it should be noted that in SP HNC patients, who mostly (76%) presented tumors in oropharynx, tongue or floor of the mouth (versus 45% in LRR HNC patients), it was also observed a higher rate of acute dysphagia grade 3–4 (67% versus 44% in LRR HNC patients). A further reason could be found in our definition of re-RT that, unlike many other analyses, excluded patients with overlap only in low dose volumes. Indeed, a recent study highlighted the importance to consider overlapping volumes to evaluate and compare toxicity from different studies (28). In clinical practice, these high rates of acute toxicity should be taken into consideration during the evaluation of the benefit-risk balance and the patients should be clearly informed of this risk. Among the 41 patients who received chemotherapy, twelve patients (29%) developed acute hematologic toxicity, that was fatal for one of them. Further studies are needed to better delineate the benefit and risks of concurrent chemotherapy. With a rate of new grade ≥3 late toxicities equal to 15%, our study presented similar results to those found in the literature, which report a range of grade ≥3 late toxicities from 14.2% to 57.1% (18). Although acute dysphagia ≥3 was common, persistent re-RT-related grade 3–4 dysphagia was observed in only two of the 41 evaluable patients (5%), thus being in the range from 1.7%–24% found in other analyses (26, 33, 36). The only other grade ≥3 toxicity was osteoradionecrosis (10%), which presented comparable incidence to that of other studies (13, 35), but it was more common than in many other analyses reporting rates between 2.6%–7.1% (19, 21, 22, 24, 36). However, in our study, the limited number of patients should be taken into account when assessing late toxicity. Moreover, a direct comparison with other studies is often not possible, due to different cohort characteristics and different definitions used both for re-RT and toxicity, as well as the diversity in the duration of the follow-up.

For patients with recurrent or metastatic HNC, therapeutic options have recently been improved with the advent of immunotherapy. The use of PD-1 inhibitors, such as pembrolizumab and nivolumab (vs. methotrexate, docetaxel, or cetuximab), led to improved overall survival, as showed in the KEYNOTE-040, KEYNOTE-048 and CheckMate 141 trials (47–49). Other recent clinical trials confirmed that the PD-1 inhibitors are a valid option in this challenging patient group (50–52). Moreover, recent and ongoing trials have evaluated the role of immunotherapy in combination with RT (53–56), also applying radiation techniques such stereotactic ablative RT (57).

The current study has some limitations. The cohort was relatively small with diversity in tumor location and tumor stage resulting in limited statistical power. Furthermore, the relatively short overall survival does not allow to draw long-term considerations. Notably, the true incidence of late toxicity may be underestimated. However, these limitations need to be considered along with the important strengths and novelties of the present analysis. Firstly, it was conducted using a prospectively collected database. In the evaluation of toxicity, this also allowed for baseline dysfunction resulting from previous treatments or tumors being considered. Indeed, compared to other studies, a unique feature of the present study is the careful and prospective data collection of toxicity, which can give a more realistic and accurate picture of their true incidence. Furthermore, in all the patients the treatment protocol consisted of the same dose/fractionation schedule and, if chemotherapy was possible, a homogenous therapy regimen was administered throughout the years. This important strength can relativize the fact that the patients were treated over a wide period, since the treatment protocol remained the same. A further strength of the study was the long follow-up of the patients which made it possible to collect valuable information about late toxicity and tumor progression. Finally, the clear definition of re-RT and the report of overlapping volumes represent a strength and allow comparisons with other studies.

## 5 Conclusions

This study showed that hyperfractionated 60 Gy re-RT plus platinum-based chemotherapy was a feasible treatment option with acceptable toxicity for carefully selected LRR/SP patients. Patients with a Charlson comorbidity index ≥3 had a higher probability of not completing the treatment resulting in unsatisfactory benefits from re-RT. T-and N-stage, disease-free interval and baseline dysfunction in the head and neck area should be also considered in the decision making. Further studies are needed to investigate the role of chemotherapy and immune checkpoint inhibitors combined with re-RT.

## Data availability statement

The original contributions presented in the study are included in the article/**Supplementary Material**. Further inquiries can be directed to the corresponding author.

## Ethics statement

The studies involving human participants were reviewed and approved by Ethics Committee of the Brandenburg Medical School “Theodor Fontane” (MHB) (E-01-20220110, approval date 25.01.2022). Written informed consent for participation was not required for this study in accordance with the national legislation and the institutional requirements.

## Author contributions

Conceptualization: AB, CSco and CSch. Methodology: AB and AF. Software: CSco, RF and PW. Formal analysis: CSco. Investigation, AF, CSch and DZ. Data curation: CSco. Writing—original draft preparation: CSco. Writing—review and editing: CSco, CSch, AB, DZ, AF, RF and SB. Visualization: CSco, DZ and PW. Supervision: AB, CSch, DZ and SB. project administration: AB. All authors contributed to the article and approved the submitted version.
