# Supplementary material for: Re-irradiation for head and neck cancer: outcome and toxicity analysis using a prospective single institution database
Source: Front Oncol. 2023 Jun 29;13:1175609. doi: 10.3389/fonc.2023.1175609 (PMC10346436; doi:10.3389/fonc.2023.1175609)
Supplement: Supplementary file 1 [file DataSheet_1.docx]

Supplementary Material

**Re-irradiation for Head and Neck Cancer: Outcome and Toxicity Analysis Using a Prospective Single Institution Database**

Chiara Scolari, André Buchali, Achim Franzen, Robert Förster, Paul Windisch, Stephan Bodis, Daniel R. Zwahlen and Christina Schröder

*** Correspondence:** Chiara Scolari: [chiara.scolari@uzh.ch](mailto:chiara.scolari@uzh.ch)

# Supplementary Figures and Tables

## Supplementary Tables

Supplementary Table 1. Tumor and treatment characteristics at the first radiotherapy course (n=61).

|  |  |  | **Median (IQR) or N (%)** |
| --- | --- | --- | --- |
| Demographics | Age at start of first RT (y) | | 56.5 (48.1–62.2) |
|  | Smoking | Never | 4 (6.6) |
|  |  | Former (>6 months before RT) | 9 (14.8) |
|  |  | Current | 38 (62.3) |
|  |  | Unknown | 10 (16.4) |
| First diagnosis | Site | Oropharynx | 24 (39.3) |
|  |  | Tongue/floor of the mouth | 13 (21.3) |
|  |  | Hypopharynx | 9 (14.8) |
|  |  | Larynx | 7 (11.5) |
|  |  | Neck only | 4 (6.6) |
|  |  | Nasopharynx | 2 (3.3) |
|  |  | Other | 2 (3.3) |
|  | Histology | Squamous cell carcinoma | 62 (100.0) |
|  | T stage | T0 | 5 (8.2) |
|  |  | T1 | 12 (19.7) |
|  |  | T2 | 15 (24.6) |
|  |  | T3 | 17 (27.9) |
|  |  | T4 | 12 (19.7) |
|  | N stage | N0 | 23 (37.7) |
|  |  | N1 | 9 (14.8) |
|  |  | N2 | 28 (45.9) |
|  |  | N3 | 1 (1.6) |
|  | Other HNC before diagnosis of HNC treated with RT | | 7 (11.5) |
| First treatment | Surgery | Both primary site + lymph node | 38 (77.6)* |
|  |  | Only primary site | 6 (12.2)* |
|  |  | Only lymph node | 5 (10.2)* |
|  |  | R0 | 32 (65.3)* |
|  |  | R1 | 14 (28.6)* |
|  |  | R2 | 3 (6.1)* |
|  |  | Extracapsular extension | 11 (22.4)* |
|  | Chemotherapy | Not indicated | 34 (55.7) |
|  |  | Indicated but not applied | 9 (14.8) |
|  |  | Patient refusal | 2 |
|  |  | Age | 3 |
|  |  | Comorbidity | 3 |
|  |  | Unknown | 1 |
|  |  | Early terminated | 6 (9.8) |
|  |  | Deterioration of general condition | 1 |
|  |  | Comorbidity | 1 |
|  |  | Toxicity of chemotherapy | 3 |
|  |  | Patient’s will | 1 |
|  |  | Applied planned treatment | 12 (19.7) |
|  |  | Neoadjuvant | 1 (5.6)^†^ |
|  |  | Concurrent alone | 6 (33.3)^†^ |
|  |  | Concurrent and adjuvant | 11 (61.1)^†^ |
|  |  | Cis-5FU | 9 (50.0)^†^ |
|  |  | Carbo-5FU | 6 (33.3)^†^ |
|  |  | From Cis-5FU switch to Carbo-5FU | 2 (11.1)^†^ |
|  |  | Cis/Vinorelbine | 1 (5.6)^†^ |

*Abbreviations: RT, radiotherapy; HNC, head and neck cancer; Cis-5FU, cisplatin plus fluorouracil; Carbo-5FU, carboplatin plus fluorouracil; Cis, cisplatin. *Percentage of postoperative patients (N=49). ^†^Percentage of those receiving chemotherapy (N=18).*

Supplementary Table 2. Frequency of Comorbid Disease per Charlson Index in the entire cohort (n=61)

| Disease per Charlson Index (N=61) | n | % |
| --- | --- | --- |
| Myocardial infarction | 14 | 23.0 |
| Congestive heart failure | 3 | 4.9 |
| Peripheral vascular disease | 6 | 9.8 |
| Cerebrovascular disease | 1 | 1.6 |
| Dementia | 3 | 4.9 |
| Chronic pulmonary disease | 10 | 16.4 |
| Connective tissue disease | – | – |
| Peptic ulcer disease | 10 | 16.4 |
| Mild liver disease | 3 | 4.9 |
| Diabetes mellitus without complications | 11 | 18.0 |
| Diabetes mellitus with complications | – | – |
| Hemiplegia | – | – |
| Renal disease | 11 | 18.0 |
| Cancer | 61 | 100.0 |
| Leukemia | – | – |
| Lymphoma | – | – |
| Moderate or severe liver disease | – | – |
| Metastatic carcinoma | – | – |
| AIDS/HIV | – | – |

Supplementary Table 3. Distribution of patient, tumor and treatment characteristics between subgroups of patients divided according to the presentation type. Outcomes of univariate logistic regression analysis with presentation type as dependent variable (second primary was the outcome of interest vs. locoregional recurrence) and one possible predictor as independent variable.

|  | Presentation type | |  |  |
| --- | --- | --- | --- | --- |
|  | LRR (N=40)  n (%)* | SP (N=21)  n (%)* | OR  (95% CI) | P |
| rT stage (4 vs. 0–3) |  |  | 0.614  (0.211–1.781) | .369 |
| T0 (n=10) | 10 (25.0) | – |  |  |
| T1 (n=3) | 2 (5.0) | 1 (4.8) |  |  |
| T2 (n=10) | 3 (7.5) | 7 (33.3) |  |  |
| T3 (n=7) | 3 (7.5) | 4 (19.0) |  |  |
| T4 (n=31) | 22 (55.0) | 9 (42.9) |  |  |
| rN stage (2–3 vs. 0–1) |  |  | 1.250  (0.426–3.664) | .684 |
| N0 (n=33) | 23 (57.5) | 10 (47.6) |  |  |
| N1 (n=4) | 2 (5.0) | 2 (9.5) |  |  |
| N2 (n=22) | 13 (32.5) | 9 (42.9) |  |  |
| N3 (n=2) | 2 (5.0) | – |  |  |
| Recurrent site |  |  |  |  |
| Oropharynx (n=18) | 10 (25.0) | 8 (38.1) |  |  |
| Tongue/floor of the mouth (16) | 8 (20.0) | 8 (38.1) |  |  |
| Hypopharynx (n=3) | 1 (2.5) | 2 (9.5) |  |  |
| Larynx (n=8) | 6 (15) | 2 (9.5) |  |  |
| Neck only (n=10) | 10 (100.0) | – |  |  |
| Nasopharynx (n=3) | 2 (5.0) | 1 (4.8) |  |  |
| Other (n=3) | 3 (7.7) | – |  |  |
| Disease-free interval (>24 vs. ≤24) |  |  | 44.786  (8.433–237.8) | **<.001** |
| ≤24 months (n=35) | 33 (82.5) | 2 (9.5) | Ref. |  |
| >24 months (n=26) | 7 (17.5) | 19 (90.5) |  |  |
| Charlson comorbidity (≥3 vs. 1–2) |  |  | 2.043  (0.697–5.985) | .193 |
| 1 (n=18) | 14 (35.0) | 4 (19.0) |  |  |
| 2 (n=18) | 12 (30.0) | 6 (28.6) |  |  |
| ≥3 (n=25) | 14 (35.0) | 11 (52.4) |  |  |
| Baseline Dysphagia (3–4 vs. 0–2) |  |  | 0.580  (0.175–1.919) | .373 |
| Grade 0–2 (n=42) | 26 (65.0) | 16 (76.2) | Ref. |  |
| Grade 3–4 (n=19) | 14 (35.0) | 5 (23.8) |  |  |
| Age at start of re-RT (y) (>60 vs. ≤60) |  |  | 1.344  (0.466–3.877) | .584 |
| ≤60 years (n=32) | 22 (55.0) | 10 (47.6) | Ref. |  |
| >60 years (n=29) | 18 (45.0) | 11 (52.4) |  |  |
| Surgery (Yes vs. No) |  |  | 1.216  (0.422–3.501) | .717 |
| No (n=31) | 21 (52.5) | 10 (47.6) | Ref. |  |
| Yes (n=30) | 19 (47.5) | 11 (52.4) |  |  |
| Chemotherapy |  |  |  |  |
| Indicated but not applied (n=16) | 10 (26.3) | 6 (31.6) | Ref. | .598 |
| Early terminated (n=17) | 13 (34.2) | 4 (21.1) | 0.513  (0.113–2.322) | .386 |
| Completed as planned (n=24) | 15 (39.5) | 9 (47.4 | 1.000  (0.271–3.694) | 1.000 |
| Re-RT dose (Gy) (<60 vs. 60) |  |  | 1.167  (0.376–3.617) | .789 |
| 60 Gy (n=42) | 28 (70.0) | 14 (66.7) | Ref. |  |
| <60 Gy (n=19) | 12 (30.0) | 7 (33.3) |  |  |

*Abbreviations: OR, odds ratio; CI, confidence interval; SP, second primary; LRR, locoregional recurrence. * Percentage refers to presentation type.*

Supplementary Table 4. Distribution of patient, tumor and treatment characteristics between subgroups of patients divided according to Charlson comorbidity index. Univariate logistic regression analysis with Charlson comorbidity index as dependent variable (index ≥3 as outcome of interest vs. index of 1–2) and one possible predictor as independent variable.

|  | **Charlson comorbidity index** | | |  |  |
| --- | --- | --- | --- | --- | --- |
|  | 1 (N=18)  n (%)* | 2 (N=18)  n (%)* | ≥3 (N=25)  n (%)* | OR  (95% CI) | P |
| rT stage (4 vs. 0–3) |  |  |  | 0.194  (0.064–0.593) | **.004** |
| T0 (n=10) | 1 (5.6) | 1 (5.6) | 8 (32.0) |  |  |
| T1 (n=3) | – | – | 3 (12.0) |  |  |
| T2 (n=10) | 2 (11.1) | 3 (16.7) | 5 (20.0) |  |  |
| T3 (n=7) | 2 (11.1) | 3 (16.7) | 2 (8.0) |  |  |
| T4 (n=31) | 13 (72.2) | 11 (61.1) | 7 (28.0) |  |  |
| rN stage (2–3 vs. 0–1) |  |  |  | 1.390  (0.490–3.941) | .536 |
| N0 (n=33) | 10 (55.6) | 11 (61.1) | 12 (48.0) |  |  |
| N1 (n=4) | – | 2 (11.1) | 2 (8.0) |  |  |
| N2 (n=22) | 7 (38.9) | 5 (27.8) | 10 (40.0) |  |  |
| N3 (n=2) | 1 (5.6) | – | 1 (4.0) |  |  |
| Recurrent site |  |  |  |  |  |
| Oropharynx (n=18) | 4 (22.2) | 5 (27.8) | 9 (36.0) |  |  |
| Tongue/floor of the mouth (n=16) | 7 (38.9) | 5 (27.8) | 4 (16.0) |  |  |
| Hypopharynx (n=3) | 1 (5.6) | 2 (11.1) | – |  |  |
| Larynx (n=8) | 4 (22.2) | 2 (11.1) | 2 (8.0) |  |  |
| Neck only (n=10) | 1 (5.6) | 1 (5.6) | 8 (32.0) |  |  |
| Nasopharynx (n=3) | 1 (5.6) | 1 (5.6) | 1 (4.0) |  |  |
| Other (n=3) | – | 2 (11.1) | 1 (4.0) |  |  |
| Presentation type (SP vs. LRR) |  |  |  | 2.043  (0.697–5.985) | .193 |
| Locoregional recurrence (n=40) | 14 (77.8) | 12 (66.7) | 14 (56.0) | Ref. |  |
| Second primary (n=21) | 4 (22.2) | 6 (33.3) | 11 (44.0) |  |  |
| Disease-free interval (mo) (>24 vs. ≤24) |  |  |  | 1.917  (0.679–5.414) | .219 |
| ≤24 months (n=35) | 11 (61.1) | 12 (66.7) | 12 (48.0) | Ref. |  |
| >24 months (n=26) | 7 (38.9) | 6 (33.3) | 13 (52.0) |  |  |
| Age at start of re-RT (y) (>60 vs. ≤60) |  |  |  | 3.145  (1.087–9.103) | **.035** |
| ≤60 (n=32) | 14 (77.8) | 9 (50.0) | 9 (36.0) | Ref. |  |
| >60 (n=29) | 4 (22.2) | 9 (50.0) | 16 (64.0) |  |  |
| Baseline dysphagia (3–4 vs. 0–2) |  |  |  | 0.778  (0.255–2.371) | .659 |
| Grade 0–2 (n=42) | 11 (61.1) | 13 (72.2) | 18 (72.0) |  |  |
| Grade 3–4 (n=19) | 7 (38.9) | 5 (27.8) | 7 (28.0) |  |  |
| Surgery |  |  |  | 1.591  (0.569–4.445) | .376 |
| No (n=31) | 10 (55.6) | 10 (55.6) | 11 (44.0) |  |  |
| Yes (n=30) | 8 (44.4) | 8 (44.4) | 14 (56.0) |  |  |
| Chemotherapy |  |  |  |  |  |
| Indicated but not applied (n=16) | 3 (17.6) | 3 (18.8) | 10 (41.7) | Ref. | .144 |
| Early terminated (n=17) | 9 (52.9) | 3 (18.8) | 5 (20.8) | 0.250  (0.058–1.070) | .062 |
| Completed as planned (n=24) | 5 (29.4) | 10 (62.5) | 9 (37.5) | 0.360  (0.097–1.330) | .125 |
| Re-RT dose (Gy) (<60 vs. 60) |  |  |  | 2.000  (0.667–6.007) | .217 |
| 60 Gy (n=42) | 12 (66.7) | 15 (83.3) | 15 (60.0) | Ref. |  |
| <60 Gy (n=19) | 6 (33.3) | 3 (16.7) | 10 (40.0) |  |  |

*Abbreviations:* *OR, odds ratio; CI, confidence interval; SP, second primary; LRR, locoregional recurrence. * Percentage refers to Charlson comorbidity index.*

Supplementary Table 5. Distribution of patient, tumor and treatment characteristics between subgroups of patients divided according to rT stage and univariate logistic regression analysis with rT stage as dependent variable (rT4 as outcome of interest vs. rT0–3) and one possible predictor as independent variable.

|  | rT stage | |  |  |
| --- | --- | --- | --- | --- |
|  | rT0–3 (N=30)  n (%)* | rT4 (N=31)  n (%)* | OR  (95% CI) | P |
| rN stage (2–3 vs. 0–1) |  |  | 0.544  (0.192–1.540) | .252 |
| N0 (n=33) | 14 (46.7) | 19 (61.3) |  |  |
| N1 (n=4) | 2 (6.7) | 2 (6.5) |  |  |
| N2 (n=22) | 13 (43.3) | 9 (29.0) |  |  |
| N3 (n=2) | 1 (3.3) | 1 (3.2) |  |  |
| Recurrent site |  |  |  |  |
| Oropharynx (n=18) | 10 (33.3) | 8 (25.8) |  |  |
| Tongue/floor of the mouth (16) | 6 (20.0) | 10 (32.3) |  |  |
| Hypopharynx (n=3) | 3 (10.0) | – |  |  |
| Larynx (n=8) | – | 8 (25.8) |  |  |
| Neck only (n=10) | 10 (33.3) | – |  |  |
| Nasopharynx (n=3) | 1 (3.3) | 2 (6.5) |  |  |
| Other (n=3) | – | 3 (9.6) |  |  |
| Presentation type (SP vs. LRR) |  |  | 0.614  (0.211–1.781) | .369 |
| Locoregional recurrence (n=40) | 18 (60.0) | 22 (71.0) | Ref. |  |
| Second primary (n=21) | 12 (40.0) | 9 (29.0) |  |  |
| Disease-free interval (>24 vs. ≤24) |  |  | 0.722  (0.261–1.998) | .530 |
| ≤24 months (n=35) | 16 (53.3) | 19 (61.3) | Ref. |  |
| >24 months (n=26) | 14 (46.7) | 12 (38.7) |  |  |
| Charlson comorbidity (≥3 vs. 1–2) |  |  | 0.194  (0.064–0.593) | **.004** |
| 1 (n=18) | 5 (16.7) | 13 (41.9) |  |  |
| 2 (n=18) | 7 (23.3) | 11 (35.5) |  |  |
| ≥3 (n=25) | 18 (60.0) | 7 (22.6) |  |  |
| Baseline Dysphagia (3–4 vs. 0–2) |  |  | 1.512  (0.507–4.515) | .458 |
| Grade 0–2 (n=42) | 22 (73.3) | 20 (64.5) |  |  |
| Grade 3–4 (n=19) | 8 (26.7) | 11 (35.5) | Ref. |  |
| Age at start of re-RT (y) (>60 vs. ≤60) |  |  | 0.632  (0.230–1.738) | .374 |
| ≤60 years (n=32) | 14 (46.7) | 18 (58.1) | Ref. |  |
| >60 years (n=29) | 16 (53.3) | 13 (41.9) |  |  |
| Surgery (Yes vs. No) |  |  | 0.552  (0.200–1.524) | .252 |
| No (n=31) | 13 (43.3) | 18 (58.1) | Ref. |  |
| Yes (n=30) | 17 (56.7) | 13 (41.9) |  |  |
| Chemotherapy |  |  |  |  |
| Indicated but not applied (n=16) | 8 (28.6) | 8 (27.6) | Ref. | .354 |
| Early terminated (n=17) | 6 (21.4) | 11 (37.9) | 1.833  (0.454–7.408) | .395 |
| Completed as planned (n=24) | 14 (50.0) | 10 (34.5) | 0.714  (0.200–2.549) | .604 |
| Re-RT dose (Gy) (<60 vs. 60) |  |  | 1.512  (0.507–4.515) | .458 |
| 60 Gy (n=42) | 22 (73.3) | 20 (64.5) | Ref. |  |
| ≥50 Gy and <60 Gy (n=10) | 7 (23.3) | 3 (9.7) |  |  |
| <50 Gy (n=9) | 1 (3.3) | 8 (25.8) |  |  |

*Abbreviations: OR, odds ratio; CI, confidence interval; SP, second primary; LRR, locoregional recurrence. * Percentage refers to rT stage.*

Supplementary Table 6. Distribution of patient, tumor and treatment characteristics between subgroups of patients divided according to re-RT dose and univariate logistic regression analysis with re-RT dose as dependent variable (<60 Gy as outcome of interest vs. 60 Gy) and one possible predictor as independent variable.

|  | **Re-RT dose** | | |  |  |
| --- | --- | --- | --- | --- | --- |
|  | 60 Gy  (N=42)  n (%)* | ≥50 Gy and <60 Gy  (N=10)  n (%)* | <50 Gy  (N=9)  n (%)* | OR  (95% CI) | P |
| rT stage (4 vs. 0–3) |  |  |  | 1.512  (0.507–4.515) | .458 |
| T0 (n=10) | 7 (16.7) | 2 (20.0) | 1 (11.1) |  |  |
| T1 (n=3) | 1 (2.4) | 2 (20.0) | – |  |  |
| T2 (n=10) | 9 (21.4) | 1 (10.0) | – |  |  |
| T3 (n=7) | 5 (11.9) | 2 (20.0) | – |  |  |
| T4 (n=31) | 20 (47.6) | 3 (30.0) | 8 (88.9) |  |  |
| rN stage (2–3 vs. 0–1) |  |  |  | 3.067  (0.999–9.413) | **.050** |
| N0 (n=33) | 26 (61.9) | 4 (40.0) | 3 (33.3) |  |  |
| N1 (n=4) | 3 (7.1) | 1 (10.0) | – |  |  |
| N2 (n=22) | 12 (28.6) | 4 (40.0) | 6 (66.7) |  |  |
| N3 (n=2) | 1 (2.4) | 1 (10.0) | – |  |  |
| Recurrent site |  |  |  |  |  |
| Oropharynx (n=18) | 10 (23.8) | 4 (40.0) | 4 (44.4) |  |  |
| Tongue/floor of the mouth (n=16) | 13 (31.0) | 2 (20.0) | 1 (11.1) |  |  |
| Hypopharynx (n=3) | 3 (7.1) | – | – |  |  |
| Larynx (n=8) | 4 (9.5) | 1 (10.0) | 3 (33.3) |  |  |
| Neck only (n=10) | 7 (16.7) | 2 (20.0) | 1 (11.1) |  |  |
| Nasopharynx (n=3) | 3 (7.1) | – | – |  |  |
| Other (n=3) | 2 (4.8) | 1 (10.0) | – |  |  |
| Presentation type (SP vs. LRR) |  |  |  | 1.167  (0.376–3.617) | .789 |
| Locoregional recurrence (n=40) | 28 (66.7) | 6 (60.0) | 6 (66.7) | Ref. |  |
| Second primary (n=21) | 14 (33.3) | 4 (40.0) | 3 (33.3) |  |  |
| Disease-free interval (mo) (>24 vs. ≤24) |  |  |  | 0.706  (0.232–2.148) | .540 |
| ≤24 months (n=35) | 23 (54.8) | 6 (60.0) | 6 (66.7) | Ref. |  |
| >24 months (n=26) | 19 (45.2) | 4 (40.0) | 3 (33.3) |  |  |
| Charlson comorbidity (≥3 vs. 1–2) |  |  |  | 2.000  (0.676–6.007) | .217 |
| 1 (n=18) | 12 (28.6) | 1 (10.0) | 5 (55.6) |  |  |
| 2 (n=18) | 15 (35.7) | – | 3 (33.3) |  |  |
| ≥3 (n=25) | 15 (35.7) | 9 (90.0) | 1 (11.1) |  |  |
| Age at start of re-RT (y) (>60 vs. ≤60) |  |  |  | 0.990  (0.334–2.930) | .986 |
| ≤60 (n=32) | 22 (52.4) | 5 (50.0) | 5 (55.6) | Ref. |  |
| >60 (n=29) | 20 (47.6) | 5 (50.0) | 4 (44.4) |  |  |
| Baseline Dysphagia (3–4 vs. 0–2) |  |  |  | 1.458  (0.463–4.595) | .519 |
| Grade 0–2 (n=42) | 30 (71.4) | 5 (50.0) | 7 (77.8) | Ref. |  |
| Grade 3–4 (n=19) | 12 (28.6) | 5 (50.0) | 2 (22.2) |  |  |
| Surgery (Yes vs. No) |  |  |  | 2.286  (0.750–6.968) | .146 |
| No (n=31) | 24 (57.1) | 2 (20.0) | 5 (55.6) | Ref. |  |
| Yes (n=30) | 18 (42.9) | 8 (80.0) | 4 (44.4) |  |  |
| Chemotherapy |  |  |  |  |  |
| Indicated but not applied (n=16) | 11 (27.5) | 3 (30.0) | 2 (28.6) | Ref. | .126 |
| Early terminated (n=17) | 9 (22.5) | 3 (30.0) | 5 (71.4) | 1.956  (0.471–8.114) | .356 |
| Completed as planned (n=24) | 20 (50.0) | 4 (40.0) | – | 0.440  (0.098–1.985) | .286 |

*Abbreviations:* *SP, second primary; LRR, locoregional recurrence. * Percentage refers to re-RT dose.*

Supplementary Table 7. Characteristics of the R0-resected patients (N=5).

|  | **Subject 9** | **Subject 14** | **Subject 15** | **Subject 20** | **Subject 67** |
| --- | --- | --- | --- | --- | --- |
| Presence of a tumor prior to the diagnosis of the one treated with 1. RT | No | No | No | No | No |
| Initial tumor |  |  |  |  |  |
| Site | Oropharynx | Floor of mouth | Oropharynx | Oropharynx | Hypopharynx |
| T stage | 1 | 2 | 2 | 2 | 4 |
| N stage | 0 | 0 | 2 | 0 | 2 |
| Initial treatment |  |  |  |  |  |
| Surgery (R-status) | Yes (R1) | Yes (R0) | Yes (R0) | Yes (R0) | Yes (R0) |
| Chemotherapy | No | No | No | No | No |
|  | (not ind.) | (not ind.) | (not ind.) | (not ind.) | (not ind.) |
| Dose of first RT (Gy) | 70 | 56 | 64 | 56 | 72 |
| Presence of a tumor prior to diagnosis of the one treated with re-RT | Yes | No | No | No | No |
| Previous surgery for a tumor that arose prior to the one treated with re-RT | Yes | No | No | No | No |
| Recurrent tumor |  |  |  |  |  |
| Disease-free interval (months) | 108.8 | 4.8 | 5.5 | 4.8 | 220.0 |
| Presentation type | SP | LRR | SP | LRR | SP |
| Recurrent site | Larynx | Floor of mouth | Floor of mouth | Neck only | Oropharynx |
| rT stage | 4 | 1 | 2 | 0 | 4 |
| rN stage | 2 | 0 | 2 | 2 | 0 |
| Overall cancer stage | IV | I | IV | IV | IV |
| Age at start of re-RT (years) | 55 | 63 | 48 | 66 | 67 |
| Charlson comorbidity score | 1 | 4 | 2 | 2 | 5 |
| Baseline dysphagia before re-RT | 1 | 3 | 3 | 0 | 3 |
| Re-treatment |  |  |  |  |  |
| Surgery (R-status) | Yes (R0) | Yes (R0) | Yes (R0) | Yes (R0) | Yes (R0) |
| Chemotherapy | Yes | Yes | Yes | Yes | No |
|  | (early term.) | (as planned) | (as planned) | (early term.) | (ind. not adm.) |
| Re-RT dose (Gy) | 20.4 | 55.2 | 60 | 60 | 50.4 |
| Overlap ≥50 Gy isodose (cm^3^) | – | 262.2 | 115.9 | 297.1 | Unknown* |
| Overlap ≥60 Gy isodose (cm^3^) | – | – | 9.2 | – | Unknown* |
| Tumor progress |  |  |  |  |  |
| Locoregional | No | No | Yes | No | No |
| Distant | No | No | No | No | No |
| Grade ≥3 toxicity | No | No | No | No | No |
| Follow-up (months) | 0.43 | 10.68 | 23.84 | 133.41 | 8.55 |
| Status | dead | dead | dead | dead | dead |
| Cause of death | Tumor-unrelated | Tumor-unrelated | Tumor-related | Tumor-unrelated | Tumor-unrelated |

*Abbreviations: HNC, head and neck cancer; RT, radiotherapy; ind., indicated; term., terminated; LRR, locoregional recurrence; SP, second primary; adm., administered; re-RT, re-irradiation. *For patients receiving their first course of RT before 2006 or outside the institution, dose plans were not available electronically.*

Supplementary Table 8. Distribution of patient, tumor and treatment characteristics between subgroups of patients divided according to the performance of surgery and the resection margin status. Univariate logistic regression analysis with surgery as dependent variable (surgery as outcome of interest vs. no surgery) and one possible predictor as independent variable.

|  |  | | | Surgery | | OR  (95%) | P |
| --- | --- | --- | --- | --- | --- | --- | --- |
|  | Resection margin | | | Yes | No |  |  |
|  | R0 | R1 | R2 | R0–2 |  |  |  |
|  | N=5  n, (%)* | N=14  n, (%)* | N=11  n, (%)* | N=30  n, (%)* | N=31  n, (%)* |  |  |
| Presence of a tumor prior to diagnosis of the one treated with first RT |  |  |  |  |  |  |  |
| No (n=54) | 5 (100.0) | 11 (78.6) | 10 (90.9) | 26 (86.7) | 28 (90.3) |  |  |
| Yes (n=7) | – | 3 (21.4) | 1 (9.1) | 4 (13.3) | 3 (9.7) |  |  |
| Presence of a tumor prior to diagnosis of the one treated with re-RT |  |  |  |  |  |  |  |
| No (n=48) | 4 (80.0) | 11 (78.6) | 9 (81.8) | 24 (80.0) | 24 (77.4) |  |  |
| Yes (n=13) | 1 (20.0) | 3 (21.4) | 2 (18.2) | 6 (20.0) | 7 (22.6) |  |  |
| Recurrent tumor |  |  |  |  |  |  |  |
| rT stage (4 vs. 0–3) |  |  |  |  |  | 0.552  (0.200–1.524) | .252 |
| T0 (n=10) | 1 (20.0) | 2 (14.3) | 3 (27.3) | 6 (20.0) | 4 (12.9) |  |  |
| T1 (n=3) | 1 (20.0) | 1 (7.1) | – | 2 (6.7) | 1 (3.2) |  |  |
| T2 (n=10) | 1 (20.0) | 1 (7.1) | 3 (27.3) | 5 (16.7) | 5 (16.1) |  |  |
| T3 (n=7) | – | 4 (28.6) | – | 4 (13.3) | 3 (9.7) |  |  |
| T4 (n=31) | 2 (40.0) | 6 (42.9) | 5 (45.4) | 13 (43.3) | 18 (58.1) |  |  |
| rN stage (2–3 vs. 0–1) |  |  |  |  |  | 1.837  (0.649–5.199) | .252 |
| N0 (n=33) | 2 (40.0) | 8 (57.1) | 4 (36.4) | 14 (46.7) | 19 (61.3) |  |  |
| N1 (n=4) | – | 1 (7.1) | 1 (9.1) | 2 (6.7) | 2 (6.5) |  |  |
| N2 (n=22) | 3 (60.0) | 5 (35.7) | 4 (36.4) | 12 (40.0) | 10 (32.3) |  |  |
| N3 (n=2) | – | – | 2 (18.2) | 2 (6.7) | – |  |  |
| Overall stage (IV vs. I–III) |  |  |  |  |  | 1.125  (0.367–3.451) | .837 |
| I (n=3) | 1 (20.0) | 1 (7.1) | – | 2 (6.7) | 1 (3.2) |  |  |
| II (n=7) | – | 1 (7.1) | 2 (18.2) | 3 (10.0) | 4 (12.9) |  |  |
| III (n=7) | – | 2 (14.3) | 1 (9.1) | 3 (10.0) | 4 (12.9) |  |  |
| IV (n=44) | 4 (80.0) | 10 (71.4) | 8 (72.7) | 22 (73.3) | 22 (71.0) |  |  |
| Recurrent site |  |  |  |  |  |  |  |
| Oropharynx | 1 (20.0) | 5 (35.7) | 3 (27.3) | 9 (30.0) | 9 (29.0) |  |  |
| Tongue/floor of the mouth | 2 (40.0) | 6 (42.9) | 3 (27.3) | 11 (36.7) | 5 (16.1) |  |  |
| Hypopharynx | – | – | – | – | 3 (9.7) |  |  |
| Larynx | 1 (20.0) | 1 (7.1) | 1 (9.1) | 3 (10.0) | 5 (16.1) |  |  |
| Neck only | 1 (20.0) | 2 (14.3) | 3 (27.3) | 6 (20.0) | 4 (12.9) |  |  |
| Nasopharynx | – | – | – | – | 3 (9.7) |  |  |
| Other | – | – | 1 (9.1) | 1 (3.3) | 2 (6.4) |  |  |
| Presentation type (SP vs. LRR) |  |  |  |  |  | 1.216  (0.422–3.501) | .717 |
| Locoregional recurrence (n=40) | 2 (40.0) | 8 (57.1) | 9 (81.8) | 19 (63.3) | 21 (67.7) | Ref. |  |
| Second primary (n=21) | 3 (60.0) | 6 (42.9) | 2 (18.2) | 11 (36.7) | 10 (32.3) |  |  |
| Disease-free interval (>24 vs. ≤24) |  |  |  |  |  | 0.810  (0.293–2.238) | .684 |
| ≤24 months (n=35) | 3 (60.0) | 7 (50.0) | 8 (72.7) | 18 (60.0) | 17 (54.8) | Ref. |  |
| >24 months (n=26) | 2 (40.0) | 7 (50.0) | 3 (27.3) | 12 (40.0) | 14 (45.2) |  |  |
| Re-treatment |  |  |  |  |  |  |  |
| Chemotherapy |  |  |  |  |  |  |  |
| Indicated but not adm. (n=16) | 1 (20.0) | 6 (42.9) | 6 (54.5) | 13 (43.3) | 3 (9.7) | Ref. | .020 |
| Early terminated (n=17) | 2 (40.0) | 2 (14.3) | 4 (35.4) | 8 (26.7) | 9 (29.0) | 0.205  (0.042–0.992) | .049 |
| Adm. as planned (n=24) | 2 (40.0) | 5 (35.7) | 1 (9.1) | 8 (26.7) | 16 (51.6) | 0.115  (0.025–0.525) | **.005** |
| Re-RT dose (Gy) (<60 Gy vs. 60 Gy) |  |  |  |  |  | 2.286  (0.750–6.968) | .146 |
| <50 (n=9) | 1 (20.0) | 1 (7.1) | 2 (18.2) | 4 (13.3) | 5 (16.1) |  |  |
| ≥50 and <60 (n=10) | 2 (40.0) | 3 (21.4) | 3 (27.3) | 8 (26.7) | 2 (6.5) |  |  |
| 60 Gy (n=41) | 2 (40.0) | 10 (71.4) | 6 (54.5) | 18 (60.0) | 24 (77.4) |  |  |
| Charlson comorbidity (≥3 vs. 1–2) |  |  |  |  |  | 1.591  (0.569–4.445) | .376 |
| 1 (n=18) | 1 (20.0) | 4 (28.6) | 3 (27.3) | 8 (26.7) | 10 (32.3) |  |  |
| 2 (n=18) | 2 (40.0) | 3 (21.4) | 3 (27.3) | 8 (26.7) | 10 (32.3) |  |  |
| 3 (n=14) | – | 3 (21.4) | 4 (36.4) | 7 (23.3) | 11 (35.5) |  |  |
| 4 (n=6) | 1 (20.0) | 1 (7.1) | – | 2 (6.7) | 7 (22.6) |  |  |
| 5 (n=4) | 1 (20.0) | 3 (21.4) | – | 4 (13.3) | – |  |  |
| 6 (n=1) | – | – | 1 (9.1) | 1 (3.3) | – |  |  |
| Age at start of re-RT (y) | 63.1  (51.5–66.7) | 58.4  (51.6–73.8) | 60.7  (55.0–65.7) | 59.8  (54.7–66.5) | 58.7  (50.5–70.3) |  |  |
| Baseline Dysphagia (3–4 vs. 0–2) |  |  |  |  |  | 1.664  (0.557–4.974) | .362 |
| Grade 0–2 (n=42) | 2 (40.0) | 9 (64.3) | 8 (72.7) | 19 (63.3) | 23 (74.2) |  |  |
| Grade 3–4 (n=19) | 3 (60.0) | 5 (35.7) | 3 (27.3) | 11 (36.7) | 8 (25.8) | Ref. |  |
| Age at start of re-RT (y) (>60 vs. ≤60) |  |  |  |  |  | 0.933  (0.342–2.551) | .893 |
| <50 (n=10) | 1 (20.0) | 3 (21.4) | 1 (9.1) | 5 (16.7) | 5 (16.1) |  |  |
| ≥50 and <60 (n=19) | 1 (20.0) | 5 (35.7) | 2 (18.2) | 8 (26.7) | 11 (35.5) |  |  |
| ≥60 and <70 (n=18) | 3 (60.0) | 2 (14.3) | 6 (54.5) | 11 (36.7) | 7 (22.6) |  |  |
| ≥70 and <80 (n=11) | – | 2 (14.3) | 1 (9.1) | 3 (10.0) | 8 (25.8) |  |  |
| ≥80 (n=3) | – | 2 (14.3) | 1 (9.1) | 3 (10.0) | – |  |  |
| Status at last follow-up |  |  |  |  |  |  |  |
| Alive (n=9) | – | 4 (28.6) | 1 (9.1) | 5 (16.7) | 4 (12.9) |  |  |
| HNC-related death (n=36) | 1 (20.0) | 9 (64.3) | 7 (63.6) | 17 (56.7) | 19 (61.3) |  |  |
| HNC-unrelated death (n=16) | 4 (80.0) | 1 (7.1) | 3 (27.3) | 8 (26.7) | 8 (25.8) |  |  |

*Abbreviation: OR, odds ratio; CI, confidence interval; HNC, head and neck cancer; LRR, locoregional recurrence; SP, second primary; adm., administered. * Percentage refers to performance of surgery.*

Supplementary Table 9. Distribution of patient, tumor and treatment characteristics between subgroups of patients divided according to chemotherapy and report of p-value from chi-square test.

|  | **Chemotherapy*** | | |  |
| --- | --- | --- | --- | --- |
|  | indicated but not administered  (N=16)  n (%)^†^ | early  terminated (N=17)  n (%)^†^ | administered  as planned (N=24)  n (%)^†^ | P |
| rT stage (4 vs. 0–3) |  |  |  | .346 |
| T0 (n=10) | 2 (12.5) | 4 (23.5) | 4 (16.7) |  |
| T1 (n=3) | – | – | 3 (12.5) |  |
| T2 (n=8) | 4 (25.0) | – | 4 (16.7) |  |
| T3 (n=7) | 2 (12.5) | 2 (11.8) | 3 (12.5) |  |
| T4 (n=29) | 8 (50.0) | 11 (64.7) | 10 (41.7) |  |
| rN stage (2–3 vs. 0–1) |  |  |  | .417 |
| N0 (n=31) | 11 (68.8) | 7 (41.2) | 13 (54.2) |  |
| N1 (n=3) | – | 1 (5.9) | 2 (8.3) |  |
| N2 (n=21) | 5 (31.3) | 7 (41.2) | 9 (37.5) |  |
| N3 (n=2) | – | 2 (11.8) | – |  |
| Recurrent site |  |  |  |  |
| Oropharynx (n=16) | 7 (43.8) | 3 (17.6) | 6 (25.0) |  |
| Tongue/floor of the mouth (n=14) | 4 (25.0) | 3 (17.6) | 7 (29.2) |  |
| Hypopharynx (n=3) | – | – | 3 (12.5) |  |
| Larynx (n=8) | 2 (12.5) | 4 (23.5) | 2 (8.3) |  |
| Neck only (n=10) | 2 (12.5) | 4 (23.5) | 4 (16.7) |  |
| Nasopharynx (n=3) | – | 2 (11.8) | 1 (4.2) |  |
| Other (n=3) | 1 (6.3) | 1 (5.9) | 1 (4.2) |  |
| Presentation type (SP vs. LRR) |  |  |  | .592 |
| Locoregional recurrence (n=38) | 10 (62.5) | 13 (76.5) | 15 (62.5) |  |
| Second primary (n=19) | 6 (37.5) | 4 (23.5) | 9 (37.5) |  |
| Disease-free interval (mo) (>24 vs. ≤24) |  |  |  | .787 |
| ≤24 months (n=33) | 9 (56.3) | 11 (64.7) | 13 (54.2) |  |
| >24 months (n=24) | 7 (43.8) | 6 (35.3) | 11 (45.8) |  |
| Charlson comorbidity |  |  |  | **.051** |
| 1 (n=17) | 3 (18.8) | 9 (52.9) | 5 (20.8) |  |
| 2 (n=16) | 3 (18.8) | 3 (17.6) | 10 (41.7) |  |
| ≥3 (n=24) | 10 (62.5) | 5 (29.4) | 9 (37.5) |  |
| Age at start of re-RT (y) (>60 vs. ≤60) |  |  |  | .076 |
| ≤60 (n=30) | 5 (31.3) | 12 (70.6) | 13 (54.2) |  |
| >60 (n=27) | 11 (68.8) | 5 (29.4) | 11 (45.8) |  |
| Baseline Dysphagia (3–4 vs. 0–2) |  |  |  | .654 |
| Grade 0–2 (n=39) | 10 (62.5) | 11 (64.7) | 18 (75.0) |  |
| Grade 3–4 (n=18) | 6 (37.5) | 6 (35.3) | 6 (25.0) |  |
| Surgery (Yes vs. No) |  |  |  | **.011** |
| No (n=28) | 3 (18.8) | 9 (52.9) | 16 (66.7) |  |
| Yes (n=29) | 13 (81.3) | 8 (47.1) | 8 (33.3) |  |
| Re-RT dose (Gy) (<60 vs. 60) |  |  |  | .110 |
| 60 Gy (n=40) | 11 (68.8) | 9 (52.9) | – |  |
| <60 Gy (n=17) | 5 (31.3) | 8 (47.1) | 4 (16.7) |  |
| Status at last follow-up |  |  |  |  |
| Alive (n=9) | 2 (12.5) | 1 (5.9) | 6 (25.0) |  |
| HNC-related death (n=33) | 10 (62.5) | 12 (70.6) | 11 (45.8) |  |
| HNC-unrelated death (n=15) | 4 (25.0) | 4 (23.5) | 7 (29.2) |  |

*Abbreviations: SP, second primary; LRR, locoregional recurrence. * Patients who received Cetuximab (n=3) and one patient without indication for chemotherapy, were excluded from the analysis. ^†^ Percentage refers to chemotherapy.*

Supplementary Table 10. Characteristics regarding the patients, who survived more than nine years.

|  | **Subject 1** | **Subject 20** | **Subject 25** | **Subject 35** | **Subject 37** | **Subject 45** |
| --- | --- | --- | --- | --- | --- | --- |
| Follow-up (months) | 123.3 | 133.4 | 154.1 | 118.5 | 128.9 | 107.0 |
| Initial tumor |  |  |  |  |  |  |
| T stage | 2 | 2 | 4 | 2 | 1 | 2 |
| N stage | 2 | 0 | 2 | 2 | 1 | 0 |
| Initial treatment |  |  |  |  |  |  |
| Surgery (R-status) | Yes (R1) | Yes (R0) | No | Yes (R0) | Yes (R0) | Yes (R0) |
| Chemotherapy | No | No | Yes | No | No | No |
|  | (not ind.) | (not ind.) | (as planned) | (pat. refusal) | (not ind.) | (not ind.) |
| Dose of first RT (Gy) | 56 | 56 | 72 | 56 | 56 | 56 |
| Recurrent tumor |  |  |  |  |  |  |
| Disease-free interval (months) | 37.1 | 4.8 | 39.5 | 27.3 | 63.6 | 8.9 |
| Presentation type | LRR | LRR | LRR | LRR | SP | SP |
| Recurrent site | Oropharynx | Neck only | Neck only | Oropharynx | Hypopharynx | Floor of mouth |
| rT stage | 3 | 0 | 0 | 3 | 2 | 4 |
| rN stage | 0 | 2 | 2 | 0 | 0 | 1 |
| Overall cancer stage | III | IV | IV | III | II | IV |
| Age at start of re-RT (years) | 44 | 66 | 60 | 71 | 64 | 58 |
| Charlson comorbidity index | 1 | 2 | 3 | 2 | 1 | 2 |
| Baseline dysphagia before re-RT | 2 | 0 | 2 | 1 | 2 | 2 |
| Re-treatment |  |  |  |  |  |  |
| Surgery (R-status) | Yes (R1) | Yes (R0) | No | No | No | Yes (R1) |
| Chemotherapy | Yes | Yes | Yes | Yes | Yes | Yes |
|  | (as planned) | (early term.) | (as planned) | (as planned) | (as planned) | (as planned) |
| Re-RT dose (Gy) | 60 | 60 | 60 | 60 | 60 | 60 |
| Overlap ≥50 Gy isodose (cm^3^) | Unknown* | 297.1 | 111.5 | 261.8 | Unknown* | 239.1 |
| Overlap ≥60 Gy isodose (cm^3^) | Unknown* | – | 33.3 | – | Unknown* | – |
| Tumor progress |  |  |  |  |  |  |
| Locoregional | no | no | no | no | no | no |
| Distant | no | no | no | no | no | no |
| Grade ≥3 toxicity | ORN | no | no | no | no | ORN |

*Abbreviations: RT, radiotherapy; ind., indicated; pat., patient; term., terminated; LRR, locoregional recurrence; SP, second primary; re-RT, re-irradiation. *For patients receiving their first course of RT before 2006 or outside the institution, dose plans were not available electronically.*

Supplementary Table 11. Progression-free survival (PFS) outcomes of the entire cohort and of different groups of patients divided according to the completion of treatment (completion of re-RT with ≥50 Gy, with 60 Gy or with 60 Gy plus partial or complete chemotherapy). Further divisions of each group regarding chemotherapy, surgery and MIRI RPA classification were performed.

|  | 1-year PFS | 2-year PFS | 5-year PFS | PFS in months | HR | P |  |
| --- | --- | --- | --- | --- | --- | --- | --- |
|  | % | % | % | median (IQR) | (95% CI) |  |  |
|  |  |  |  |  |  |  |  |
| **All patients (N=61)** | 25.3 | 17.5 | 13.6 | 6.3 (3.0–14.1) |  |  |  |
|  |  |  |  |  |  |  |  |
| Completion of the intended treatment (No vs. Yes) | | | | | 2.994  (1.561–5.743) | <.001 |  |
| No (n=41) | 13.4 | 5.4 | 2.7 | 6.0 (1.3–10.7) |  |  |  |
| Yes (n=20)* | 50.0 | 43.8 | 37.5 | 9.8 (5.1–154.1) | Ref. |  |  |
| Chemotherapy |  |  |  |  |  |  |  |
| No, although indicated (n=16) | 6.3 | 0 | 0 | 6.0 (3.0–8.3) | Ref. | .004 |  |
| Early terminated (n=17) | 15.7 | 15.7 | 7.8 | 3.0 (1.2–6.9) | 0.927  (0.453–1.898) | .836 |  |
| Yes, as planned (n=24) | 49.7 | 34.8 | 29.8 | 10.7 (5.1–118.5) | 0.339  (0.165–0.700) | .003 |  |
| Re-RT completion (<50 vs. ≥50) |  |  |  |  | 40.649  (12.5–132.0) | <.001 |  |
| No, re-RT dose <50 Gy (n=9) | 0 | 0 | 0 | 0.6 (0.4–0.9) |  |  |  |
| Yes (n=52) | 29.7 | 20.6 | 16.0 | 7.9 (4.6–19.7) | Ref. |  |  |
| ≥50 Gy and <60 Gy (n=10) | 20.0 | 0 | 0 | 8.3 (3.1–10.8) |  |  |  |
| 60 Gy (n=42) | 32.3 | 26.4 | 20.5 | 7.1 (4.9–24.7) |  |  |  |
| Surgery (Yes vs. No) |  |  |  |  | 0.956  (0.556–1.642) | .869 |  |
| No (n=31) | 21.8 | 21.8 | 14.5 | 5.8 (3.1–11.8) | Ref. |  |  |
| Yes (n=30) | 29.1 | 12.5 | 12.5 | 7.1 (2.3–14.1) |  |  |  |
| MIRI RPA classification |  |  |  |  |  |  |  |
| Class I (n=13) | 30.8 | 7.7 | 7.7 | 8.8 (6.9–14.1) | Ref. | .324 |  |
| Class II (n=35) | 27.2 | 27.2 | 20.4 | 6.0 (3.1–24.7) | 0.990  (0.500–1.961) | .978 |  |
| Class III (n=13) | 15.4 | 0 | 0 | 5.0 (1.2–8.3) | 1.642  (0.732–3.681) | .229 |  |
|  |  |  |  |  |  |  |  |
| **Patients who completed re-RT with ≥50 Gy (N=52)** | 29.7 | 20.6 | 16.0 | 7.9 (4.6–5.8) |  |  |  |
|  |  |  |  |  |  |  |  |
| Chemotherapy |  |  |  |  |  |  |  |
| No, although indicated (n=14) | 7.1 | 0 | 0 | 6.3 (4.9–8.5) | Ref. | .231 |  |
| Early terminated (n=12) | 22.2 | 22.2 | 11.1 | 6.2 (2.3–11.8) | 0.862  (0.414–1.794) | .691 |  |
| Yes, as planned (n=24) | 49.7 | 34.8 | 29.8 | 10.7 (5.1–118.5) | 1.674  (0.706–3.968) | .242 |  |
| Surgery |  |  |  |  | 0.976  (0.539–1.769) | .937 |  |
| No (n=26) | 26.0 | 26.0 | 17.3 | 6.3 (4.6–24.7) | Ref. |  |  |
| Yes (n=26) | 33.6 | 14.4 | 14.4 | 8.5 (5.0–16.9) |  |  |  |
| MIRI RPA classification |  |  |  |  |  |  |  |
| Class I (n=12) | 33.3 | 8.3 | 8.3 | 8.8 (6.9–14.1) |  |  |  |
| Class II (n=29) | 32.9 | 32.9 | 24.7 | 7.1 (4.6–34.8) | Ref. | .231 |  |
| Class III (n=11) | 18.2 | 0 | 0 | 5.1 (2.3–10.7) | 0.862  (0.414–1.794) | .691 |  |
|  |  |  |  |  | 1.674  (0.706–3.968) | .242 |  |
| **Patients who completed re-RT with 60 Gy as in protocol (N=42)** | 52.3 | 29.0 | 20.3 | 7.1 (4.9–24.7) |  |  |  |
|  |  |  |  |  |  |  |  |
| Chemotherapy |  |  |  |  |  |  |  |
| No, although indicated (n=11) | 9.1 | 0 | 0 | 6.0 (3.8–7.9) | Ref. | .024 |  |
| Early terminated (n=9) | 29.6 | 29.6 | 14.8 | 6.9 (4.1–24.7) | 0.526  (0.204–1.361) | .185 |  |
| Yes, as planned (n=20) | 50.0 | 43.8 | 37.5 | 9.8 (5.1–154.1) | 0.006  (0.302–0.128) | .006 |  |
| Surgery |  |  |  |  | 0.898  (0.452–1.782) | .758 |  |
| No (n=24) | 28.1 | 28.1 | 18.8 | 6.3 (4.6–24.7) | Ref. |  |  |
| Yes (n=18) | 38.1 | 22.9 | 22.9 | 7.9 (5.0–21.4) |  |  |  |
| MIRI RPA classification |  |  |  |  |  |  |  |
| Class I (n=8) | 25.0 | 12.5 | 12.5 | 7.9 (3.0–16.5) | Ref. | .492 |  |
| Class II (n=27) | 35.3 | 35.3 | 26.5 | 8.7 (4.6–118.5) | 0.778  (0.327–1.849) | .570 |  |
| Class III (n=7) | 28.6 | 0 | 0 | 5.1 (4.9–21.4) | 1.344  (0.447–4.043) | .598 |  |
|  |  |  |  |  |  |  |  |
| **Patients who completed re-RT with 60 Gy and who received partial or complete chemotherapy (N=29)** | 44.0 | 39.6 | 30.8 | 9.8 (5.1–118.5) |  |  |  |
|  |  |  |  |  |  |  |  |
| Surgery |  |  |  |  | 0.520  (0.190–1.428) | .205 |  |
| No (n=20) | 34.3 | 34.3 | 22.9 | 6.5 (4.3–34.8) |  |  |  |
| Yes (n=9) | 66.7 | 50.0 | 50.0 | 21.4 (8.8–133.4) |  |  |  |
|  |  |  |  |  |  |  |  |

*Abbreviations: PFS, overall survival; IQR, interquartile range; HR, hazards ratio; CI, confidence interval; re-RT, re-irradiation; MIRI RPA, recursive partitioning analysis classification in analogy to the definition by the Multi-Institution Reirradiation. *Patients who received chemotherapy as planned and definitive or adjuvant re-RT with 60 Gy.*

Supplementary Table 12. Locoregional control (LRC) outcomes of the entire cohort and of different groups of patients divided according to the completion of treatment (completion of re-RT with ≥50 Gy, with 60 Gy or with 60 Gy plus partial or complete chemotherapy). Further divisions of each group regarding chemotherapy, surgery and MIRI RPA classification were performed.

|  | 1-year LRC | 2-year LRC | 5-year LRC | LRC in months | HR | P |  |
| --- | --- | --- | --- | --- | --- | --- | --- |
|  | % | % | % | median (IQR) | (95% CI) |  |  |
|  |  |  |  |  |  |  |  |
| **All patients (N=61)** | 39.5 | 30.4 | 30.4 | 7.9 (4.1–>154) |  |  |  |
|  |  |  |  |  |  |  |  |
| Completion of the intended treatment (No vs. Yes) | | | | | 2.614  (1.210–5.646) | .014 |  |
| No (n=41) | 27.6 | 16.5 | 16.5 | 6.5 (3.0–14.1) |  |  |  |
| Yes (n=20)* | 60.0 | 52.5 | 52.5 | >154 (5.1–>154) | Ref. |  |  |
| Chemotherapy |  |  |  |  |  |  |  |
| No, although indicated (n=16) | 19.8 | 0 | 0 | 7.1 (3.8–11.3) | Ref. | .022 |  |
| Early terminated (n=17) | 21.8 | 21.8 | 7.8 | 6.2 (2.3–11.8) | 0.911  (0.389–2.133) | .831 |  |
| Yes, as planned (n=24) | 62.5 | 49.7 | 49.7 | 21.4 (5.1–>154) | 0.336  (0.144–0.785) | .012 |  |
| Re-RT completion (<50 vs. ≥50) |  |  |  |  | 19.813  (5.40–72.641) | <.001 |  |
| No, re-RT dose <50 Gy (n=9) | 0 | 0 | 0 | 0.9 (0.6–3.0) |  |  |  |
| Yes (n=52) | 43.1 | 33.3 | 33.3 | 7.9 (4.6–19.7) | Ref. |  |  |
| ≥50 Gy and <60 Gy (n=10) | 55.6 | 27.8 | 27.8 | 16.9 (6.2–>19.7) |  |  |  |
| 60 Gy (n=42) | 40.9 | 33.7 | 33.7 | 8.7 (5.1–>154) |  |  |  |
| Surgery (Yes vs. No) |  |  |  |  | 0.904  (0.469–1.742) | .763 |  |
| No (n=31) | 33.9 | 33.9 | 33.9 | 6.3 (4.1–>154) | Ref. |  |  |
| Yes (n=30) | 45.6 | 24.4 | 24.4 | 11.3 (3.8–21.4) |  |  |  |
| MIRI RPA classification |  |  |  |  |  |  |  |
| Class I (n=13) | 47.6 | 23.8 | 23.8 | 11.3 (6.9–16.9) | Ref. | .577 |  |
| Class II (n=35) | 38.7 | 38.7 | 38.7 | 6.3 (3.8–>154) | 1.093  (0.480–2.492) | .832 |  |
| Class III (n=13) | 32.4 | 0 | 0 | 6.5 (2.3–21.4) | 1.614  (0.601–4.329) | .342 |  |
|  |  |  |  |  |  |  |  |
| **Patients who completed re-RT with ≥50 Gy (N=52)** | 43.1 | 33.3 | 33.3 | 8.8 (5.1–>154.1) |  |  |  |
|  |  |  |  |  |  |  |  |
| Chemotherapy |  |  |  |  |  |  |  |
| No, although indicated (n=14) | 21.2 | 0 | 0 | 7.1 (4.9–11.3) | Ref. | .046 |  |
| Early terminated (n=12) | 24.4 | 24.4 | 24.4 | 6.5 (3.0–11.8) | 0.822  (0.327–2.069) | .677 |  |
| Yes, as planned (n=24) | 62.5 | 49.7 | 49.7 | 21.4 (5.1–>154) | 0.353  (0.148–0.841) | .019 |  |
| Surgery |  |  |  |  | 0.925  (0.457–1.875) | .830 |  |
| No (n=26) | 37.6 | 37.6 | 37.6 | 6.5 (4.9–>154) | Ref. |  |  |
| Yes (n=26) | 49.1 | 26.3 | 26.3 | 11.3 (6.0–>133) |  |  |  |
| MIRI RPA classification |  |  |  |  |  |  |  |
| Class I (n=12) | 47.6 | 23.8 | 23.8 | 11.3 (6.9–16.9) | Ref. | .511 |  |
| Class II (n=29) | 43.9 | 43.9 | 43.9 | 8.7 (5.1–>154) | 0.899  (0.383–2.110) | .807 |  |
| Class III (n=11) | 35.4 | 0 | 0 | 6.5 (4.9–21.4) | 1.520  (0.548–4.220) | .421 |  |
|  |  |  |  |  |  |  |  |
| **Patients who completed re-RT with 60 Gy as in protocol (N=42)** | 40.9 | 33.7 | 33.7 | 8.7 (5.1–>154) |  |  |  |
|  |  |  |  |  |  |  |  |
| Chemotherapy |  |  |  |  |  |  |  |
| No, although indicated (n=11) | 10.6 | 0 | 0 | 6.3 (3.8–7.9) | Ref. | .024 |  |
| Early terminated (n=9) | 29.6 | 29.6 | 29.6 | 6.9 (4.1–>133.4) | 0.526  (0.204–1.361) | .185 |  |
| Yes, as planned (n=20) | 60.0 | 52.5 | 52.5 | >154 (5.1–>154) | 0.302  (0.128–0.712) | .006 |  |
| Surgery |  |  |  |  | 0.898  (0.452–1.782) | .758 |  |
| No (n=24) | 40.7 | 40.7 | 40.7 | 6.5 (4.9–>154) | Ref. |  |  |
| Yes (n=18) | 41.0 | 24.6 | 24.6 | 8.8 (6.0–21.4) |  |  |  |
| MIRI RPA classification |  |  |  |  |  |  |  |
| Class I (n=8) | 25.0 | 12.5 | 12.5 | 7.9 (3.0–11.3) | Ref. | .492 |  |
| Class II (n=27) | 47.1 | 47.1 | 47.1 | 11.8 (5.1–>154) | 0.778  (0.327–1.849) | .570 |  |
| Class III (n=7) | 35.7 | 0 | 0 | 6.5 (4.9–21.4) | 1.344  (0.447–4.043) | .598 |  |
|  |  |  |  |  |  |  |  |
| **Patients who completed re-RT with 60 Gy and who received partial or complete chemotherapy (N=29)** | 50.9 | 45.8 | 45.8 | 21.4 (5.1–>154) |  |  |  |
|  |  |  |  |  |  |  |  |
| Surgery |  |  |  |  | 0.653  (0.207–2.060) | .468 |  |
| No (n=20) | 43.8 | 43.8 | 43.8 | 8.7 (4.6–>154) |  |  |  |
| Yes (n=9) | 50.0 | 50.0 | 50.0 | 21.4 (8.8–>133) |  |  |  |
|  |  |  |  |  |  |  |  |

*Abbreviations: LRC, overall survival; IQR, interquartile range; HR, hazards ratio; CI, confidence interval; re-RT, re-irradiation; MIRI RPA, recursive partitioning analysis classification in analogy to the definition by the Multi-Institution Reirradiation. *Patients who received complete chemotherapy as planned and definitive or adjuvant re-RT with 60 Gy.*

Supplementary Table 13. Overall survival (OS) outcomes of patients with locoregional recurrence versus those with second primary tumor. Further divisions of each patient group regarding treatment completion, chemotherapy, surgery and MIRI RPA classification were performed.

|  | 1-year OS | 2-year OS | 5-year OS | OS in months | HR | P |  |
| --- | --- | --- | --- | --- | --- | --- | --- |
|  | % | % | % | median (IQR) | (95% CI) |  |  |
|  |  |  |  |  |  |  |  |
| **All patients (N=61)** | 40.6 | 19.4 | 13.6 | 10.1 (4.4–20.7) |  |  |  |
|  |  |  |  |  |  |  |  |
| **Patients with LRR HNC (N=40)** | 31.1 | 24.2 | 17.3 | 7.8 (3.7–20.7) |  |  |  |
|  |  |  |  |  |  |  |  |
| Completion of the intended treatment (No vs. Yes) | | | | | 2.787  (1.225–6.342) | **.015** |  |
| No (n=27) | 14.8 | 9.9 | 4.9 | 6.0 (2.7–10.8) |  |  |  |
| Yes (n=13)^*^ | 61.5 | 51.3 | 41.0 | 28.1 (6.3–154.1) | Ref. |  |  |
| Chemotherapy |  |  |  |  |  |  |  |
| No, although indicated (n=10) | 0 | 0 | 0 | 5.0 (4.3–8.3) | Ref. | .036 |  |
| Early terminated (n=13) | 20.5 | 20.5 | 10.3 | 5.9 (2.7–12.0) | 0.682  (0.263–1.768) | .431 |  |
| Yes, as planned (n=15) | 59.3 | 42.3 | 33.9 | 20.7 (6.3–118.5) | 0.289  (0.105–0.796) | **.016** |  |
| Re-RT completion (<60 vs. 60 Gy) |  |  |  |  | 3.357  (1.571–7.173) | **.002** |  |
| No, re-RT dose <50 Gy (n=6) | 0 | 0 | 0 | 0.9 (0.9–2.7) |  |  |  |
| Yes (n=34) | 36.5 | 28.4 | 20.3 | 10.7 (5.9–28.1) |  |  |  |
| ≥50 Gy and <60 Gy (n=6) | 16.7 | 0 | 0 | 7.8 (3.7–10.8) |  |  |  |
| 60 Gy (n=28) | 42.0 | 36.8 | 26.3 | 11.8 (5.9–118.5) | Ref. |  |  |
| Surgery (Yes vs. No) |  |  |  |  | 1.232  (0.612–2.481) | .558 |  |
| No (n=21) | 34.0 | 28.3 | 17.0 | 10.8 (3.8–28.1) | Ref. |  |  |
| Yes (n=19) | 28.4 | 18.9 | 18.9 | 6.0 (3.7–20.7) |  |  |  |
| MIRI RPA classification |  |  |  |  |  |  |  |
| Class I (n=4) | 50.0 | 25.0 | 25.0 | 6.0 (5.9–20.7) |  |  |  |
| Class II (n=24) | 38.0 | 32.6 | 21.7 | 8.3 (3.8–45.2) |  |  |  |
| Class III (n=12) | 10.0 | 10.0 | 10.0 | 5.0 (1.8–10.7) |  |  |  |
|  |  |  |  |  |  |  |  |
| **Patients with SP HNC (N=21)** | 57.1 | 14.3 | 9.5 | 13.5 (9.7–19.7) |  |  |  |
|  |  |  |  |  |  |  |  |
| Completion of the intended treatment (No vs. Yes) | | |  |  | 7.983  (1.733–36.78) | **.008** |  |
| No (n=14) | 42.9 | 0 | 0 | 10.1 (8.4–16.5) |  |  |  |
| Yes (n=7)^*^ | 85.7 | 42.9 | 0 | 23.8 (13.5–>128.9) | Ref. |  |  |
| Chemotherapy |  |  |  |  |  |  |  |
| No, although indicated (n=6) | 66.7 | 0 | 0 | 12.2 (10.1–18.4) | Ref. | .020 |  |
| Early terminated (n=4) | 25.0 | 0 | 0 | 0.5 (0.4–10.2) | 2.317  (0.608–8.826) | .218 |  |
| Yes, as planned (n=9) | 77.8 | 33.3 | 22.2 | 21.1 (13.5–34.8) | 0.230  (0.056–0.947) | **.042** |  |
| Re-RT completion (<60 vs. 60 Gy) |  |  |  |  | 3.760  (1.365–10.35) | **.010** |  |
| No, re-RT dose <50 Gy (n=3) | 0 | 0 | 0 | 0.5 (0.4–4.0) |  |  |  |
| Yes (n=18) | 66.7 | 16.7 | 11.1 | 14.8 (10.1–21.1) |  |  |  |
| ≥50 Gy and <60 Gy (n=4) | 50.0 | 0 | 0 | 9.7 (8.5–12.2) |  |  |  |
| 60 Gy (n=14) | 71.4 | 21.4 | 14.3 | 16.5 (10.2–23.8) | Ref. |  |  |
| Surgery (Yes vs. No) |  |  |  |  | 0.978  (0.394–2.429) | .962 |  |
| No (n=10) | 50.0 | 20.0 | 10.0 | 9.8 (8.4–21.1) | Ref. |  |  |
| Yes (n=11) | 63.6 | 9.1 | 9.1 | 13.5 (10.1–19.7) |  |  |  |
| MIRI RPA classification |  |  |  |  |  |  |  |
| Class I (n=9) | 55.6 | 0 | 0 | 12.2 (10.1–16.5) |  |  |  |
| Class II (n=11) | 54.5 | 27.3 | 18.2 | 14.8 (8.4–34.8) |  |  |  |
| Class III (n=1) | 100.0 | 0 | 0 | 23.8 (23.8–23.8) |  |  |  |
|  |  |  |  |  |  |  |  |

*Abbreviations: OS, overall survival; IQR, interquartile range; HR, hazards ratio; CI, confidence interval; LRR, locoregional recurrence; SP, second primary; re-RT, re-irradiation; MIRI RPA, recursive partitioning analysis classification in analogy to the definition by the Multi-Institution Reirradiation. ^*^Patients who received chemotherapy as planned and definitive or adjuvant re-RT with 60 Gy.*

Supplementary Table 14. Progression-free survival (PFS) outcomes of patients with locoregional recurrence versus those with second primary tumor. Further divisions of each patient group regarding treatment completion, chemotherapy, surgery and MIRI RPA classification were performed.

|  | 1-year PFS | 2-year PFS | 5-year PFS | PFS in months | HR | P |  |
| --- | --- | --- | --- | --- | --- | --- | --- |
|  | % | % | % | median (IQR) | (95% CI) |  |  |
|  |  |  |  |  |  |  |  |
| **All patients (N=61)** | 25.3 | 17.5 | 13.6 | 6.3 (3.0–14.1) |  |  |  |
|  |  |  |  |  |  |  |  |
| **Patients with LRR HNC (N=40)** | 23.6 | 20.3 | 16.9 | 5.1 (2.3–11.8) |  |  |  |
|  |  |  |  |  |  |  |  |
| Completion of the intended treatment (No vs. Yes) | | | | | 2.632  (1.176–5.887) | **.019** |  |
| No (n=27) | 13.0 | 8.6 | 4.3 | 5.0 (1.2–10.7) |  |  |  |
| Yes (n=13)^*^ | 46.2 | 46.2 | 46.2 | 8.7 (5.1–154.1) | Ref. |  |  |
| Chemotherapy |  |  |  |  |  |  |  |
| No, although indicated (n=10) | 0 | 0 | 0 | 3.8 (1.2–6.0) | Ref. | .014 |  |
| Early terminated (n=13) | 20.5 | 10.3 | 10.3 | 3.0 (1.3–11.8) | 0.548  (0.225–1.335) | .186 |  |
| Yes, as planned (n=15) | 45.7 | 36.6 | 36.6 | 10.7 (5.1–118.5) | 0.250  (0.098–0.639) | **.004** |  |
| Re-RT completion (<60 vs. 60 Gy) |  |  |  |  | 2.823  (1.361–5.857) | .005 |  |
| No, re-RT dose <50 Gy (n=6) | 0 | 0 | 0 | 0.8 (0.6–0.9) |  |  |  |
| Yes (n=34) | 27.8 | 23.8 | 19.9 | 6.2 (3.8–16.9) |  |  |  |
| ≥50 Gy and <60 Gy (n=6) | 16.7 | 0 | 0 | 6.2 (2.3–10.7) |  |  |  |
| 60 Gy (n=28) | 30.6 | 30.6 | 25.5 | 6.0 (3.8–118.5) | Ref. |  |  |
| Surgery (Yes vs. No) |  |  |  |  | 1.174  (0.598–2.305) | .642 |  |
| No (n=21) | 22.2 | 22.2 | 16.7 | 5.8 (3.8–11.8) | Ref. |  |  |
| Yes (n=19) | 25.3 | 16.8 | 16.8 | 5.0 (1.2–16.9) |  |  |  |
| MIRI RPA classification |  |  |  |  |  |  |  |
| Class I (n=4) | 50.0 | 25.0 | 25.0 | 3.0 (1.3–16.9) |  |  |  |
| Class II (n=24) | 26.8 | 26.8 | 21.4 | 6.0 (3.0–24.7) |  |  |  |
| Class III (n=12) | 8.3 | 8.3 | 8.3 | 5.0 (1.2–6.5) |  |  |  |
|  |  |  |  |  |  |  |  |
| **Patients with SP HNC (N=21)** | 28.6 | 14.3 | 9.5 | 8.5 (4.3–14.1) |  |  |  |
|  |  |  |  |  |  |  |  |
| Completion of the intended treatment (No vs. Yes) | | |  |  | 4.891  (1.360–17.59) | **.015** |  |
| No (n=14) | 14.3 | 0 | 0 | 6.3 (3.1–10.8) |  |  |  |
| Yes (n=7)^*^ | 57.1 | 42.9 | 28.6 | 21.4 (8.8–>128.9) | Ref. |  |  |
| Chemotherapy |  |  |  |  |  |  |  |
| No, although indicated (n=6) | 16.7 | 0 | 0 | 8.5 (7.9–11.3) | Ref. | .005 |  |
| Early terminated (n=4) | 0 | 0 | 0 | 0.4 (0.4–4.1) | 6.539  (1.317–32.46) | **.022** |  |
| Yes, as planned (n=9) | 55.6 | 33.3 | 22.2 | 19.7 (8.8–34.8) | 0.348  (0.096–1.265) | .109 |  |
| Re-RT completion (<60 vs. 60 Gy) |  |  |  |  | 2.411  (0.909–6.394) | .077 |  |
| No, re-RT dose <50 Gy (n=3) | 0 | 0 | 0 | 0.4 (0.4–0.4) |  |  |  |
| Yes (n=18) | 33.3 | 16.7 | 11.1 | 8.8 (6.3–19.7) |  |  |  |
| ≥50 Gy and <60 Gy (n=4) | 25.0 | 0 | 0 | 8.5 (3.1–10.8) |  |  |  |
| 60 Gy (n=14) | 35.7 | 21.4 | 14.3 | 8.8 (6.3–21.4) | Ref. |  |  |
| Surgery (Yes vs. No) |  |  |  |  | 0.641  (0.255–1.614) | .345 |  |
| No (n=10) | 20.0 | 20.0 | 10.0 | 4.3 (3.1–9.8) | Ref. |  |  |
| Yes (n=11) | 36.4 | 9.1 | 9.1 | 10.8 (7.9–19.7) |  |  |  |
| MIRI RPA classification |  |  |  |  |  |  |  |
| Class I (n=9) | 22.2 | 0 | 0 | 8.8 (7.9–11.3) |  |  |  |
| Class II (n=11) | 27.3 | 27.3 | 18.2 | 6.0 (3.1–34.8) |  |  |  |
| Class III (n=1) | 100.0 | 0 | 0 | 21.4 (21.4–21.4) |  |  |  |
|  |  |  |  |  |  |  |  |

*Abbreviations: PFS, progression-free survival; IQR, interquartile range; HR, hazards ratio; CI, confidence interval; LRR, locoregional recurrence; SP, second primary; re-RT, re-irradiation; MIRI RPA, recursive partitioning analysis classification in analogy to the definition by the Multi-Institution Reirradiation. ^*^Patients who received chemotherapy as planned and definitive or adjuvant re-RT with 60 Gy.*

Supplementary Table 15. Locoregional control (LRC) outcomes of patients with locoregional recurrence versus those with second primary tumor. Further divisions of each patient group regarding treatment completion, chemotherapy, surgery and MIRI RPA classification were performed.

|  | 1-year LRC | 2-year LRC | 5-year LRC | LRC in months | HR | P |  |
| --- | --- | --- | --- | --- | --- | --- | --- |
|  | % | % | % | median (IQR) | (95% CI) |  |  |
|  |  |  |  |  |  |  |  |
| **All patients (N=61)** | 39.5 | 30.4 | 30.4 | 7.9 (4.1–>154) |  |  |  |
|  |  |  |  |  |  |  |  |
| **Patients with LRR HNC (N=40)** | 34.4 | 29.5 | 29.5 | 6.2 (3.8–>154) |  |  |  |
|  |  |  |  |  |  |  |  |
| Completion of the intended treatment (No vs. Yes) | | | | | 1.781  (0.735–4.315) | .201 |  |
| No (n=27) | 27.1 | 18.1 | 18.1 | 6.2 (3.0–16.9) |  |  |  |
| Yes (n=13)^*^ | 46.2 | 46.2 | 46.2 | 8.7 (5.1–>154.1) | Ref. |  |  |
| Chemotherapy |  |  |  |  |  |  |  |
| No, although indicated (n=10) | 0 | 0 | 0 | 5.0 (3.0–7.1) | Ref. | .049 |  |
| Early terminated (n=13) | 28.4 | 28.4 | 28.4 | 6.5 (2.3–>133.4) | 0.448  (0.157–1.281) | .134 |  |
| Yes, as planned (n=15) | 53.3 | 42.7 | 42.7 | 16.9 (5.1–>154.1) | 0.277  (0.099–0.777) | **.015** |  |
| Re-RT completion (<60 vs. 60 Gy) |  |  |  |  | 1.839  (0.758–4.463) | .178 |  |
| No, re-RT dose <50 Gy (n=6) | 0 | 0 | 0 | 0.9 (0.8–>2.5) |  |  |  |
| Yes (n=34) | 37.1 | 31.8 | 31.8 | 7.1 (4.6–>154.1) |  |  |  |
| ≥50 Gy and <60 Gy (n=6) | 40.0 | 0 | 0 | 8.3 (6.2–16.9) |  |  |  |
| 60 Gy (n=28) | 36.9 | 36.9 | 36.9 | 6.5 (3.8–>154.1) | Ref. |  |  |
| Surgery (Yes vs. No) |  |  |  |  | 1.139  (0.510–2.545) | .751 |  |
| No (n=21) | 31.0 | 31.0 | 31.0 | 6.5 (4.6–>154.1) | Ref. |  |  |
| Yes (n=19) | 39.9 | 26.6 | 26.6 | 6.2 (2.3–133.4) |  |  |  |
| MIRI RPA classification |  |  |  |  |  |  |  |
| Class I (n=4) | 50.0 | 25.0 | 25.0 | 3.0 (1.3–16.9) |  |  |  |
| Class II (n=24) | 35.3 | 35.3 | 35.3 | 7.1 (3.8–>154.1) |  |  |  |
| Class III (n=12) | 24.5 | 24.5 | 24.5 | 5.1 (2.3–8.3) |  |  |  |
|  |  |  |  |  |  |  |  |
| **Patients with SP HNC (N=21)** | 48.6 | 31.2 | 31.2 | 11.3 (6.3–>128) |  |  |  |
|  |  |  |  |  |  |  |  |
| Completion of the intended treatment (No vs. Yes) | | |  |  | 10.407  (1.302–83.16) | **.027** |  |
| No (n=14) | 29.0 | 14.5 | 14.5 | 6.9 (4.1–14.1) |  |  |  |
| Yes (n=7)^*^ | 85.7 | 64.3 | 64.3 | >21.4 | Ref. |  |  |
| Chemotherapy |  |  |  |  |  |  |  |
| No, although indicated (n=6) | 44.4 | 0 | 0 | 11.3 (7.9–14.1) | Ref. | .013 |  |
| Early terminated (n=4) | 0 | 0 | 0 | 4.1 (0.4–6.9) | 6.765  (1.028–44.53) | .047 |  |
| Yes, as planned (n=9) | 77.8 | 58.3 | 58.3 | >21.4 | 0.222  (0.039–1.280) | .092 |  |
| Re-RT completion (<60 vs. 60 Gy) |  |  |  |  | 1.368  (0.359–5.212) | .646 |  |
| No, re-RT dose <50 Gy (n=3) | 0 | 0 | 0 | 3.0 (0.4–3.0) |  |  |  |
| Yes (n=18) | 53.9 | 34.6 | 34.6 | 14.1 (6.9–128.9) |  |  |  |
| ≥50 Gy and <60 Gy (n=4) | 75.0 | 75.0 | 75.0 | >19.7 |  |  |  |
| 60 Gy (n=14) | 49.0 | 30.6 | 30.6 | 11.3 (6.3–>128.9) | Ref. |  |  |
| Surgery (Yes vs. No) |  |  |  |  | 0.646  (0.206–2.026) | .453 |  |
| No (n=10) | 40.0 | 40.0 | 40.0 | 6.0 (3.1–>128.9) | Ref. |  |  |
| Yes (n=11) | 57.1 | 21.4 | 21.4 | 14.1 (8.8–21.4) |  |  |  |
| MIRI RPA classification |  |  |  |  |  |  |  |
| Class I (n=9) | 45.0 | 22.5 | 22.5 | 11.3 (7.9–14.1) |  |  |  |
| Class II (n=11) | 45.5 | 45.5 | 45.5 | 6.3 (3.1–>128.9) |  |  |  |
| Class III (n=1) | 100.0 | 0 | 0 | 21.4 (21.4–21.4) |  |  |  |
|  |  |  |  |  |  |  |  |

*Abbreviations: LRC, locoregional control; IQR, interquartile range; HR, hazards ratio; CI, confidence interval; LRR, locoregional recurrence; SP, second primary; re-RT, re-irradiation; MIRI RPA, recursive partitioning analysis classification in analogy to the definition by the Multi-Institution Reirradiation. ^*^Patients who received chemotherapy as planned and definitive or adjuvant re-RT with 60 Gy.*

Supplementary Table 16. Distribution of patient, tumor and treatment characteristics between subgroups of patients divided according to age and univariate logistic regression analysis with age as dependent variable (>60 years as outcome of interest) and one possible predictor as independent variable.

|  | **Age at start of re-RT** | |  |  |
| --- | --- | --- | --- | --- |
|  | ≤60 years (N=32)  n (%)* | >60 years (N=29)  n (%)* | OR  (95% CI) | P |
| rT stage (4 vs. 0–3) |  |  | 0.632  (0.230–1.738) | .374 |
| T0 (n=10) | 4 (12.5) | 6 (20.7) |  |  |
| T1 (n=3) | 1 (3.1) | 2 (6.9) |  |  |
| T2 (n=10) | 5 (15.6) | 5 (17.2) |  |  |
| T3 (n=7) | 4 (12.5) | 3 (10.3) |  |  |
| T4 (n=31) | 18 (56.3) | 13 (44.8) |  |  |
| rN stage (2–3 vs. 0–1) |  |  | 0.893  (0.319–2.501) | .830 |
| N0 (n=33) | 16 (50.0) | 17 (58.6) |  |  |
| N1 (n=4) | 3 (9.4) | 1 (3.4) |  |  |
| N2 (n=22) | 11 (34.4) | 11 (37.9) |  |  |
| N3 (n=2) | 2 (6.3) | – |  |  |
| Recurrent site |  |  |  |  |
| Oropharynx (n=18) | 6 (18.8) | 12 (41.4) |  |  |
| Tongue/floor of the mouth (16) | 13 (40.6) | 3 (10.3) |  |  |
| Hypopharynx (n=3) | 1 (3.1) | 2 (6.9) |  |  |
| Larynx (n=8) | 4 (12.5) | 4 (13.8) |  |  |
| Neck only (n=10) | 4 (12.5) | 6 (20.7) |  |  |
| Nasopharynx (n=3) | 3 (9.4) | – |  |  |
| Other (n=3) | 1 (3.1) | 2 (6.9) |  |  |
| Presentation type (SP vs. LRR) |  |  | 1.344  (0.466–3.877) | .584 |
| Locoregional recurrence (n=40) | 22 (68.8) | 18 (62.1) |  |  |
| Second primary (n=21) | 10 (31.3) | 11 (37.9) |  |  |
| Disease-free interval (mo) (>24 vs. ≤24) |  |  | 2.045  (0.730–5.734) | .174 |
| ≤24 months (n=35) | 21 (65.6) | 14 (48.3) | Ref. |  |
| >24 months (n=26) | 11 (34.4) | 15 (51.7) |  |  |
| Charlson comorbidity index (≥3 vs. 1–2) |  |  | 3.145  (1.087–9.103) | **.035** |
| 1 (n=18) | 14 (43.8) | 4 (13.8) |  |  |
| 2 (n=18) | 9 (28.1) | 9 (31.0) |  |  |
| ≥3 (n=25) | 9 (28.1) | 16 (55.2) |  |  |
| Baseline dysphagia (3–4 vs. 0–2) |  |  | 0.530  (0.174–1.612) | .263 |
| Grade 0–2 (n=42) | 20 (62.5) | 22 (75.9) |  |  |
| Grade 3–4 (n=19) | 12 (37.5) | 7 (24.1) |  |  |
| Surgery (Yes vs. No) |  |  | 0.933  (0.342–2.551) | .893 |
| No (n=31) | 16 (50.0) | 15 (51.7) |  |  |
| Yes (n=30) | 16 (50.0) | 14 (48.3) |  |  |
| Chemotherapy |  |  |  |  |
| Indicated but not applied (n=16) | 5 (16.7) | 11 (40.7) | Ref. | .088 |
| Early terminated (n=17) | 12 (40.0) | 5 (18.5) | 0.189  (0.043–0.836) | **.028** |
| Completed as planned (n=24) | 13 (43.3) | 11 (40.7) | 0.385  (0.102–1.451) | .158 |
| Re-RT dose (Gy) (<60 vs. 60) |  |  | 0.990  (0.334–2.930) | .986 |
| 60 Gy (n=42) | 22 (68.8) | 20 (69.0) |  |  |
| <60 Gy (n=19) | 10 (31.3) | 9 (31.0) |  |  |

*Abbreviations: OR, odds ratio; CI, confidence interval; SP, second primary; LRR, locoregional recurrence. * Percentage refers to age.*

Supplementary Table 17. Distribution of patient, tumor and treatment characteristics between subgroups of patients divided according to rN stage and univariate logistic regression analysis with rN stage as dependent variable (rN2–3 as outcome of interest) and one possible predictor as independent variable.

|  | rN stage | |  |  |
| --- | --- | --- | --- | --- |
|  | rN0–1 (N=37)  n (%)* | rN2–3 (N=24)  n (%)* | OR  (95% CI) | P |
| rT stage (4 vs. 0–3) |  |  | 0.544  (0.192–1.540) | .252 |
| T0 (n=10) | 1 (2.7) | 9 (37.5) |  |  |
| T1 (n=3) | 3 (8.1) | – |  |  |
| T2 (n=10) | 8 (21.6) | 2 (8.3) |  |  |
| T3 (n=7) | 4 (10.8) | 3 (12.5) |  |  |
| T4 (n=31) | 21 (56.8) | 10 (41.7) |  |  |
| Recurrent site |  |  |  |  |
| Oropharynx (n=18) | 14 (37.8) | 4 (16.7) |  |  |
| Tongue/floor of the mouth (16) | 10 (27.0) | 6 (25.0) |  |  |
| Hypopharynx (n=3) | 3 (8.1) | – |  |  |
| Larynx (n=8) | 4 (10.8) | 4 (16.7) |  |  |
| Neck only (n=10) | 1 (2.7) | 9 (37.5) |  |  |
| Nasopharynx (n=3) | 2 (5.4) | 1 (4.2) |  |  |
| Other (n=3) | 3 (8.1) | – |  |  |
| Presentation type (SP vs. LRR) |  |  | 1.250  (0.426–3.664) | .684 |
| Locoregional recurrence (n=40) | 25 (67.6) | 15 (62.5) | Ref. |  |
| Second primary (n=21) | 12 (32.4) | 9 (37.5) |  |  |
| Disease-free interval (>24 vs. ≤24) |  |  | 0.938  (0.331–2.652) | .903 |
| ≤24 months (n=35) | 21 (56.8) | 14 (58.3) | Ref. |  |
| >24 months (n=26) | 16 (43.2) | 10 (41.7) |  |  |
| Charlson comorbidity (≥3 vs. 1–2) |  |  | 1.390  (0.490–3.941) | .536 |
| 1 (n=18) | 5 (16.7) | 13 (41.9) |  |  |
| 2 (n=18) | 7 (23.3) | 11 (35.5) |  |  |
| ≥3 (n=25) | 18 (60.0) | 7 (22.6) |  |  |
| Baseline Dysphagia (3–4 vs. 0–2) |  |  | 0.615  (0.196–1.932) | .406 |
| Grade 0–2 (n=42) | 24 (64.9) | 18 (75.0) |  |  |
| Grade 3–4 (n=19) | 13 (35.1) | 6 (25.0) |  |  |
| Age at start of re-RT (y) (>60 vs. ≤60) |  |  | 0.893  (0.319–2.501) | .830 |
| ≤60 years (n=32) | 19 (51.4) | 13 (54.2) |  |  |
| >60 years (n=29) | 18 (48.6) | 11 (45.8) |  |  |
| Surgery (Yes vs. No) |  |  | 1.837  (0.649–5.199) | .252 |
| No (n=31) | 13 (43.3) | 18 (58.1) |  |  |
| Yes (n=30) | 17 (56.7) | 13 (41.9) |  |  |
| Chemotherapy |  |  |  |  |
| Indicated but not applied (n=16) | 11 (32.4) | 5 (21.7) | Ref. | .423 |
| Early terminated (n=17) | 8 (23.5) | 9 (39.1) | 2.475  (0.597–10.269) | .212 |
| Completed as planned (n=24) | 15 (44.1) | 9 (39.1) | 1.320  (0.345–5.050) | .685 |
| Re-RT dose (Gy) (<60 vs. 60) |  |  | 3.067  (0.999–9.413) | **.050** |
| 60 Gy (n=42) | 29 (78.4) | 13 (54.2) |  |  |
| <60 Gy (n=19) | 8 (21.6) | 11 (45.8) |  |  |

*Abbreviations: OR, odds ratio; CI, confidence interval; SP, second primary; LRR, locoregional recurrence. * Percentage refers to rN stage.*

Supplementary Table 18. Baseline, acute and late toxicities.*

|  | Grade | | | |
| --- | --- | --- | --- | --- |
|  | 1 | 2 | 3 | 4 |
| Before re-RT (n=52) (N, %) |  |  |  |  |
| Dysphagia | 5 (9.6) | 11 (21.2) | 15 (28.8) | 2 (3.8) |
| Xerostomia | 7(13.5) | 4 (7.7) | – | – |
| Dysgeusia | 3 (5.8) | 1 (1.9) | – | – |
| Fibrosis | 2 (3.8) | 10 (19.2) | – | – |
| Telangiectasia | 1 (1.9) | – | – | – |
| Worst overall | 5 (9.6) | 17 (32.7) | 15 (28.8) | 2 (3.8) |
| Acute toxicity (n=52) (N, %)^†^ |  |  |  |  |
| Dysphagia | 1 (1.9) | 5 (9.6) | 27 (51.9) | 15 (28.8) |
| Xerostomia | 20 (38.5) | 8 (15.4) | 1 (1.9) | – |
| Dysgeusia | 13 (25.0) | 16 (30.8) | – | – |
| Radiation dermatitis | 18 (34.6) | 13 (25.0) | 2 (3.8) | – |
| Mucositis | 3 (5.8) | 12 (23.1) | 24 (46.2) | – |
| Fibrosis | 2 (3.8) | 5 (9.6) | – | – |
| Telangiectasia | 1 (1.9) | 2 (3.8) | – | – |
| Worst overall | 3 (5.8) | 4 (7.7) | 28 (53.8) | 15 (28.8) |
| Late toxicity (n=41) (N, %)^†,‡^ |  |  |  |  |
| Osteoradionecrosis | – | – | 4 (9.8) | – |
| Dysphagia | 7 (17.1) | 12 (29.3) | 2 (4.9) | 5 (12.2) |
| Xerostomia | 14 (34.1) | 3 (7.3) | – | – |
| Dysgeusia | 12 (29.3) | 4 (9.8) | – | – |
| Fibrosis | 10 (24.4) | 6 (14.6) | – | – |
| Telangiectasia | 8 (19.5) | 1 (2.4) | – | – |

* Patients who terminated prematurely re-RT (N=9) were excluded. † Baseline toxicities were not subtracted. ‡ For nine of 41 evaluable patients data were unknown.

Supplementary Table 19. Acute and late re-RT-related toxicities, subtracting baseline toxicity in patients with locoregional recurrence versus those with second primary tumor.

| **Patients with locoregional recurrence (N=40)** | New grade | | | |
| --- | --- | --- | --- | --- |
| Acute toxicity (n=34) (N, %)^*^ | 1–2 | | 3–4 | |
| Dysphagia | 4 (11.8) | | 15 (44.1) | |
| Xerostomia | 10 (29.4) | | 1 (2.9) | |
| Dysgeusia | 15 (44.1) | | – | |
| Radiation dermatitis | 19 (55.9) | | 1 (2.9) | |
| Mucositis | 10 (29.4) | | 14 (35.0) | |
| Fibrosis | – | | – | |
| Telangiectasia | 1 (2.9) | | – | |
| Late toxicity (n=25) (N, %)^†^ |  |  |  |  |
| Osteoradionecrosis | – | | 2 (8.0) | |
| Dysphagia | 4 (16.0) | | 1 (4.0) | |
| Xerostomia | 4 (16.0) | | – | |
| Dysgeusia | 8 (32.0) | | – | |
| Fibrosis | 7 (28.0) | | – | |
| Telangiectasia | 2 (8.0) | | – | |
|  |  | |  | |
| **Patients with second primary tumor (N=21)** |  | |  | |
| Acute toxicity (N=18) (N, %)^*^ | 1–2 | | 3–4 | |
| Dysphagia | 1 (5.6) | | 12 (66.7) | |
| Xerostomia | 10 (55.6) | | – | |
| Dysgeusia | 10 (55.6) | | – | |
| Radiation dermatitis | 12 (66.7) | | 1 (5.6) | |
| Mucositis | 5 (27.8) | | 10 (55.6) | |
| Fibrosis | 1 (5.6) | | – | |
| Telangiectasia | 1 (5.6) | | – | |
| Late toxicity (n=16) (N, %)^†^ |  | |  | |
| Osteoradionecrosis | – | | 2 (12.5) | |
| Dysphagia | 3 (18.8) | | 1 (6.3) | |
| Xerostomia | 7 (43.8) | | – | |
| Dysgeusia | 5 (31.3) | | – | |
| Fibrosis | 7 (43.8) | | – | |
| Telangiectasia | 6 (37.5) | | – | |

*^*^ Patients who terminated prematurely re-RT were excluded (n=6 in the group of patients with LRR HNC and n=3 in the group of patients with SP HNC). ^†^ For eight of 25 LRR HNC evaluable patients and for one of 16 SP HNC evaluable patients, data were unknown.*

Supplementary Table 20. Baseline, acute and late toxicities in patients with locoregional recurrence versus those with second primary tumor.

| **LRR HNC patients (N=40)** | Grade | | | |
| --- | --- | --- | --- | --- |
|  | 1 | 2 | 3 | 4 |
| Before re-RT (n=34) (N, %)^*^ |  |  |  |  |
| Dysphagia | 4 (11.8) | 7 (20.6) | 10 (29.4) | 2 (5.9) |
| Xerostomia | 5 (14.7) | 2 (5.9) | – | – |
| Dysgeusia | 1 (2.9) | 1 (2.9) | – | – |
| Fibrosis | 1 (2.9) | 7 (20.6) | – | – |
| Telangiectasia | 2 (5.9) | – | – | – |
| Worst overall | 3 (8.8) | 12 (35.3) | 10 (29.4) | 2 (5.9) |
| Acute toxicity (n=34) (N, %)^*,†^ |  |  |  |  |
| Dysphagia | 1 (2.9) | 4 (11.8) | 15 (44.1) | 10 (29.4) |
| Xerostomia | 10 (29.4) | 5 (14.7) | 1 (2.9) | – |
| Dysgeusia | 9 (26.5) | 8 (23.5) | – | – |
| Radiation dermatitis | 12 (35.3) | 7 (20.6) | 1 (2.9) | – |
| Mucositis | 2 (5.9) | 8 (23.5) | 14 (35.0) | – |
| Fibrosis | 2 (5.9) | 3 (8.8) | – | – |
| Telangiectasia | 1 (2.9) | 2 (5.9) | – | – |
| Worst overall | 3 (8.8) | 3 (8.8) | 16 (47.1) | 10 (29.4) |
| Late toxicity (n=25) (N, %)^†,‡^ |  |  |  |  |
| Osteoradionecrosis | – | – | 2 (8.0) | – |
| Dysphagia | 3 (12.0) | 8 (32.0) | – | 2 (8.0) |
| Xerostomia | 7 (28.0) | 1 (4.0) | – | – |
| Dysgeusia | 8 (32.0) | 1 (4.0) | – | – |
| Fibrosis | 5 (20.0) | 3 (12.0) | – | – |
| Telangiectasia | 2 (8.0) | 1 (4.0) | – | – |
|  |  |  |  |  |
| **SP HNC patients (N=21)** |  |  |  |  |
| Before re-RT (n=18) (N, %)^*^ |  |  |  |  |
| Dysphagia | 1 (5.6) | 4 (22.2) | 5 (27.8) | – |
| Xerostomia | 2 (11.1) | 2 (11.1) | – | – |
| Dysgeusia | 2 (11.1) | – | – | – |
| Fibrosis | 1 (5.6) | 3 (16.7) | – | – |
| Telangiectasia | – | – | – | – |
| Worst overall | 2 (11.1) | 5 (27.8) | 5 (27.8) | – |
| Acute toxicity (n=18) (N, %)^*,†^ |  |  |  |  |
| Dysphagia | – | 1 (5.6) | 12 (66.7) | 5 (27.8) |
| Xerostomia | 10 (55.6) | 3 (16.7) | – | – |
| Dysgeusia | 4 (22.2) | 8 (44.4) | – | – |
| Radiation dermatitis | 6 (33.3) | 6 (33.3) | 1 (5.6) | – |
| Mucositis | 1 (5.6) | 4 (22.2) | 10 (55.6) | – |
| Fibrosis | – | 2 (11.1) | – | – |
| Telangiectasia | 1 (5.6) | – | – | – |
| Worst overall | – | 1 (5.6) | 12 (66.7) | 5 (27.8) |
| Late toxicity (n=16) (N, %)^†,‡^ |  |  |  |  |
| Osteoradionecrosis | – | – | 2 (12.5) | – |
| Dysphagia | 4 (25.0) | 4 (25.0) | 2 (12.5) | 3 (18.8) |
| Xerostomia | 7 (43.8) | 2 (12.5) | – | – |
| Dysgeusia | 4 (25.0) | 3 (18.8) | – | – |
| Fibrosis | 5 (31.3) | 3 (18.8) | – | – |
| Telangiectasia | 6 (37.5) | – | – | – |

*Abbreviations: LRR, locoregional recurrence; SP, second primary; HNC, head and neck cancer. ^*^ Patients who terminated prematurely re-RT were excluded (n=6 in the group of patients with LRR HNC and n=3 in the group of patients with SP HNC). † Baseline toxicities were not subtracted. ^‡^ For eight of 25 LRR HNC evaluable patients and for one of 16 SP HNC evaluable patients, data were unknown.*

## Supplementary Figures


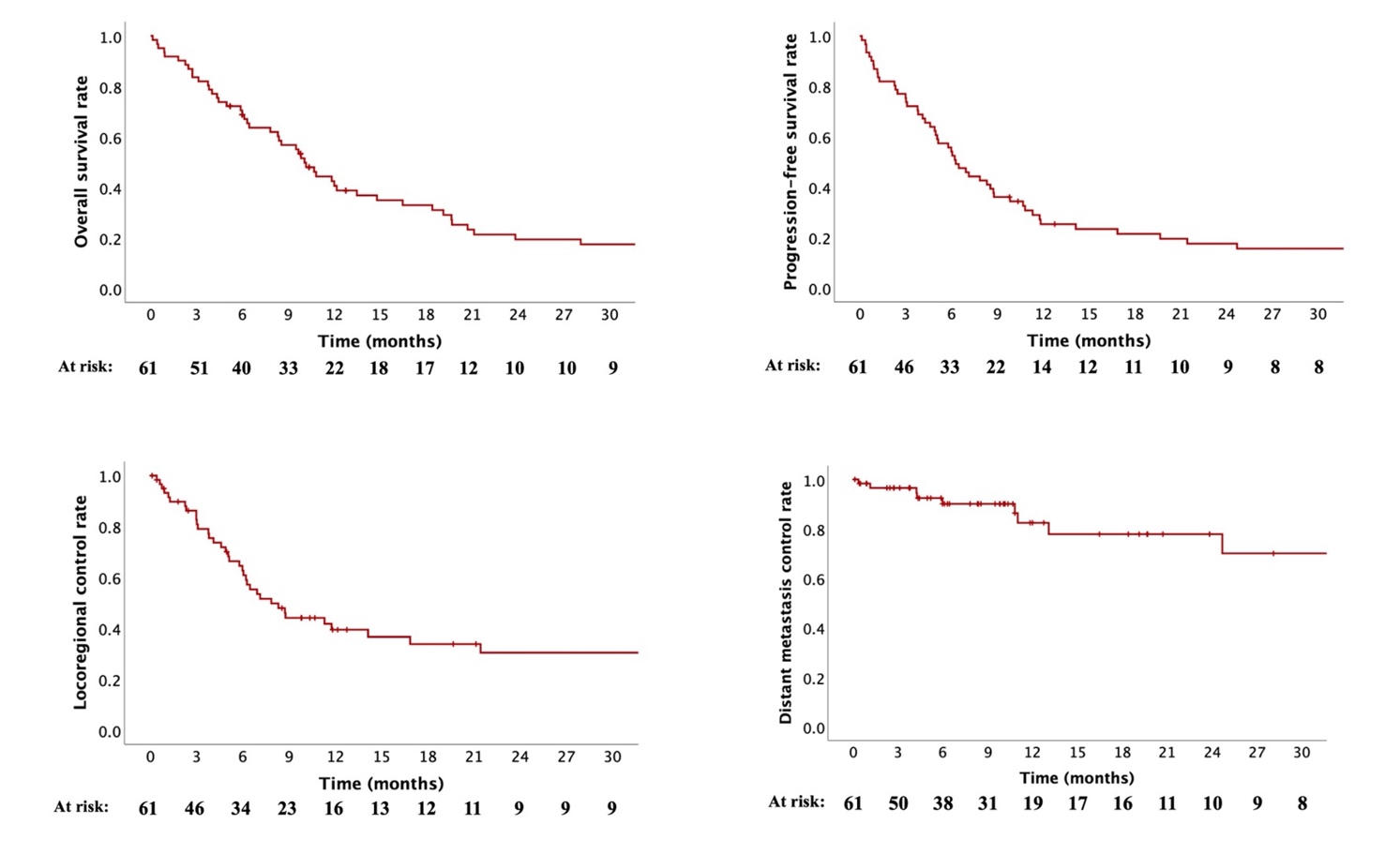


Supplementary Figure 1. Kaplan-Meier curves for (A) overall survival, (B) progression-free survival, (C) locoregional control and (D) distant metastasis control for the first 30 months after re-irradiation.


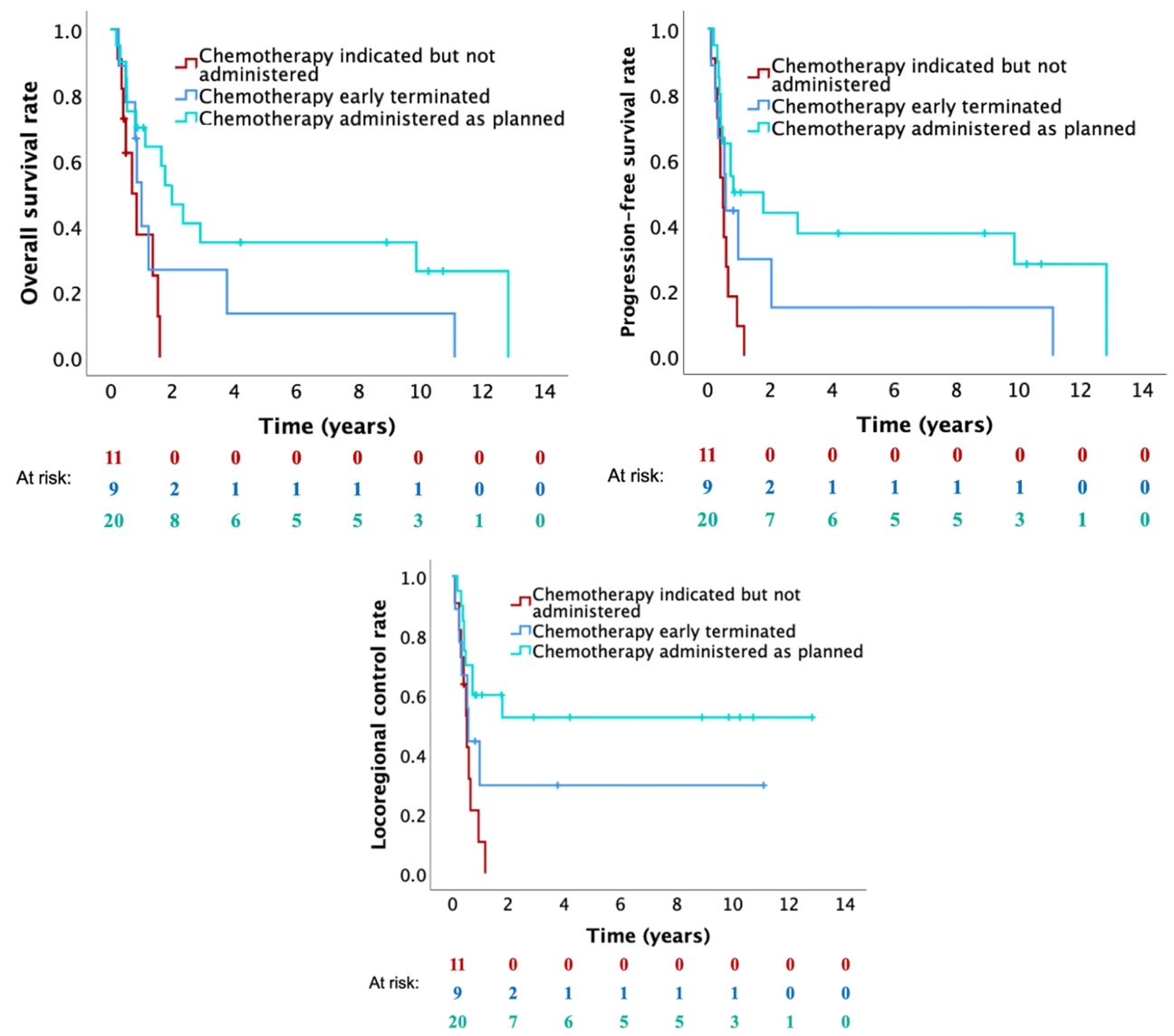


Supplementary Figure 2. Kaplan-Meier curves for (A) overall survival, (B) progression-free survival and (C) locoregional control of patients who received completed re-RT with 60 Gy as intended, further subdivided according to the administration of chemotherapy (patients for whom chemotherapy was indicated but not possible versus patients who early terminated chemotherapy versus patients who were able to complete chemotherapy).


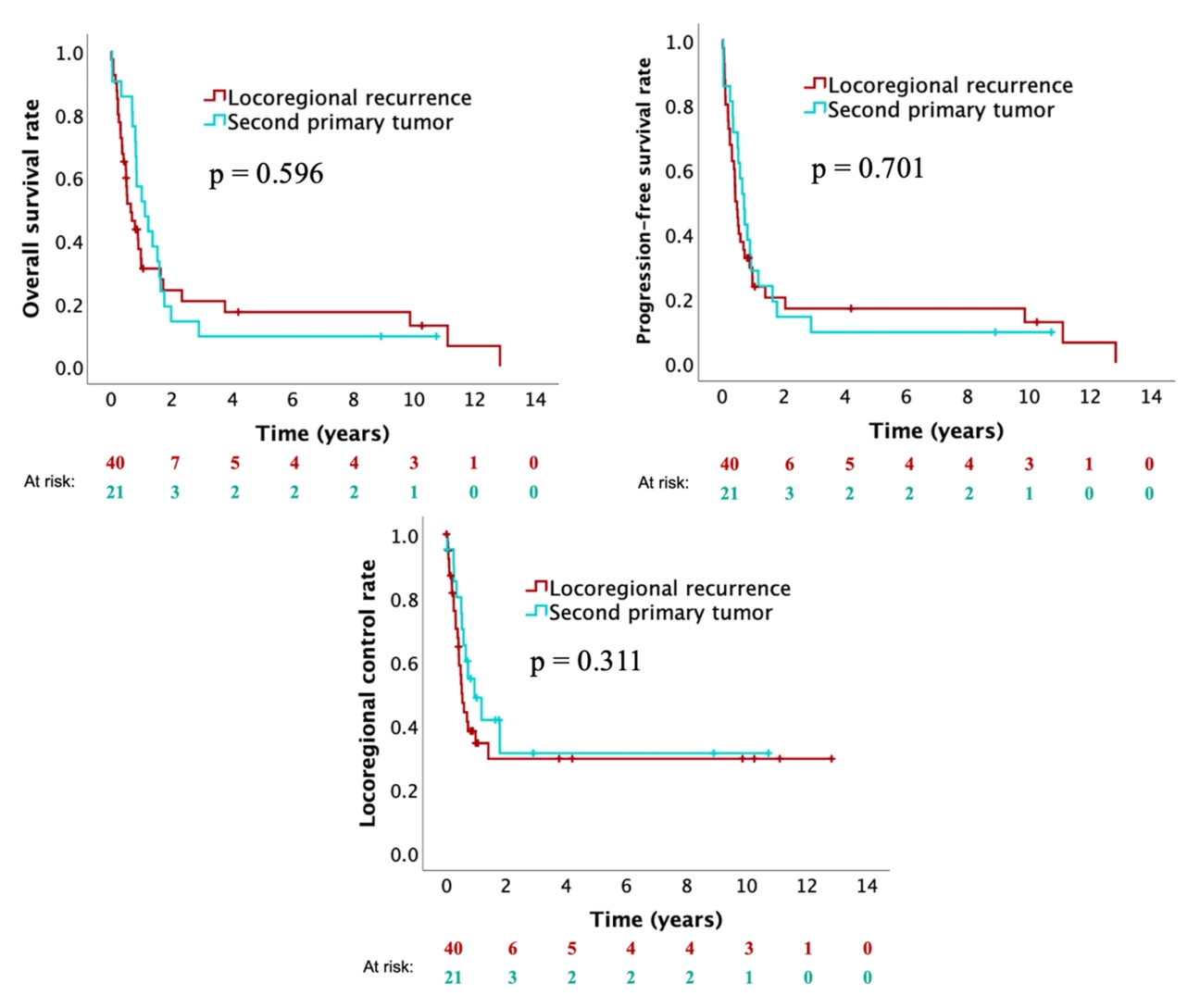


Supplementary Figure 3. Kaplan-Meier curves for (A) overall survival, (B) progression-free survival and (C) locoregional control of patients with locoregional recurrence versus patients with second primary tumor.


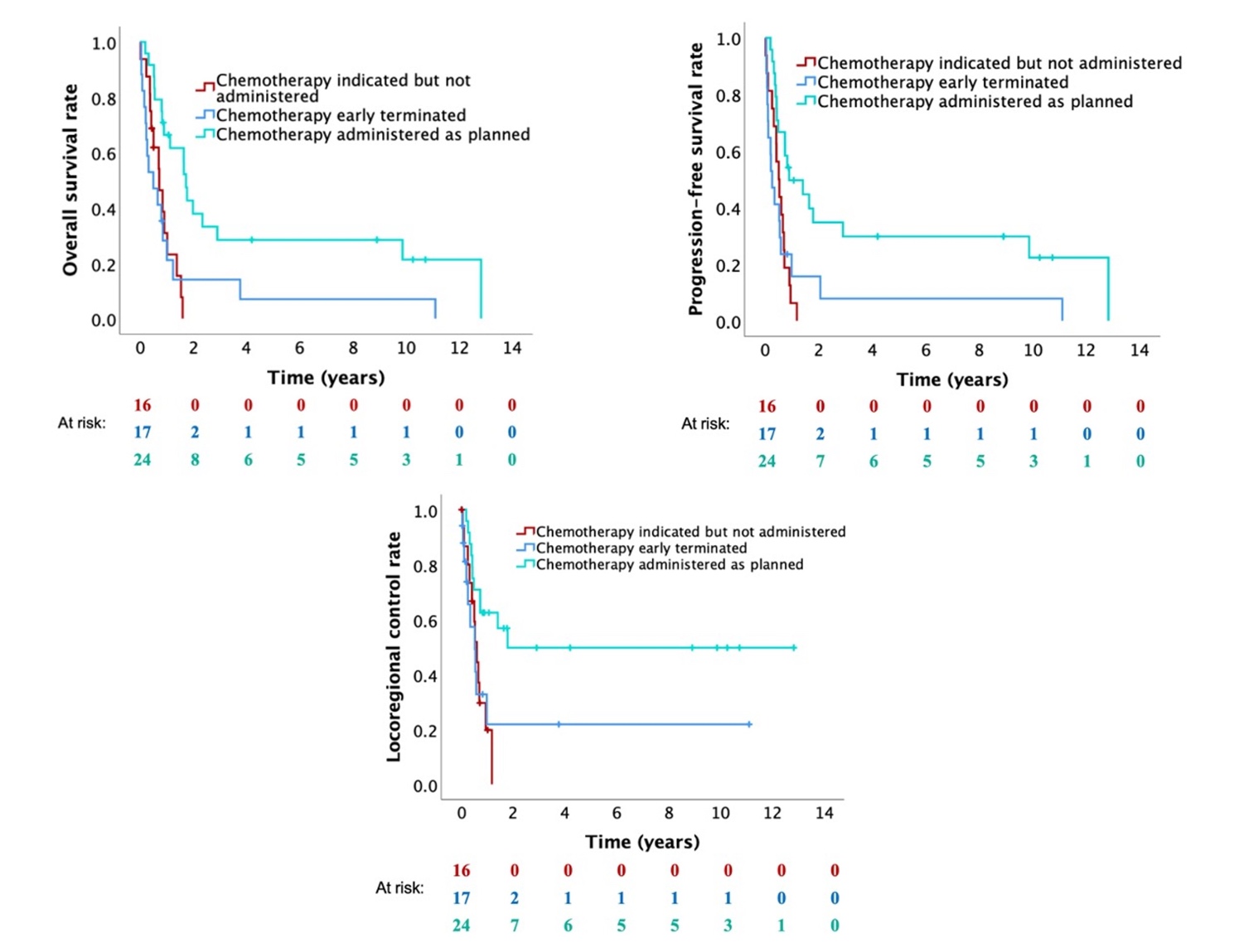


Supplementary Figure 4. Kaplan-Meier curves for (A) overall survival, (B) progression-free survival and (C) locoregional control of patients for whom chemotherapy was indicated but not possible versus patients who early terminated chemotherapy versus patients who were able to complete chemotherapy.


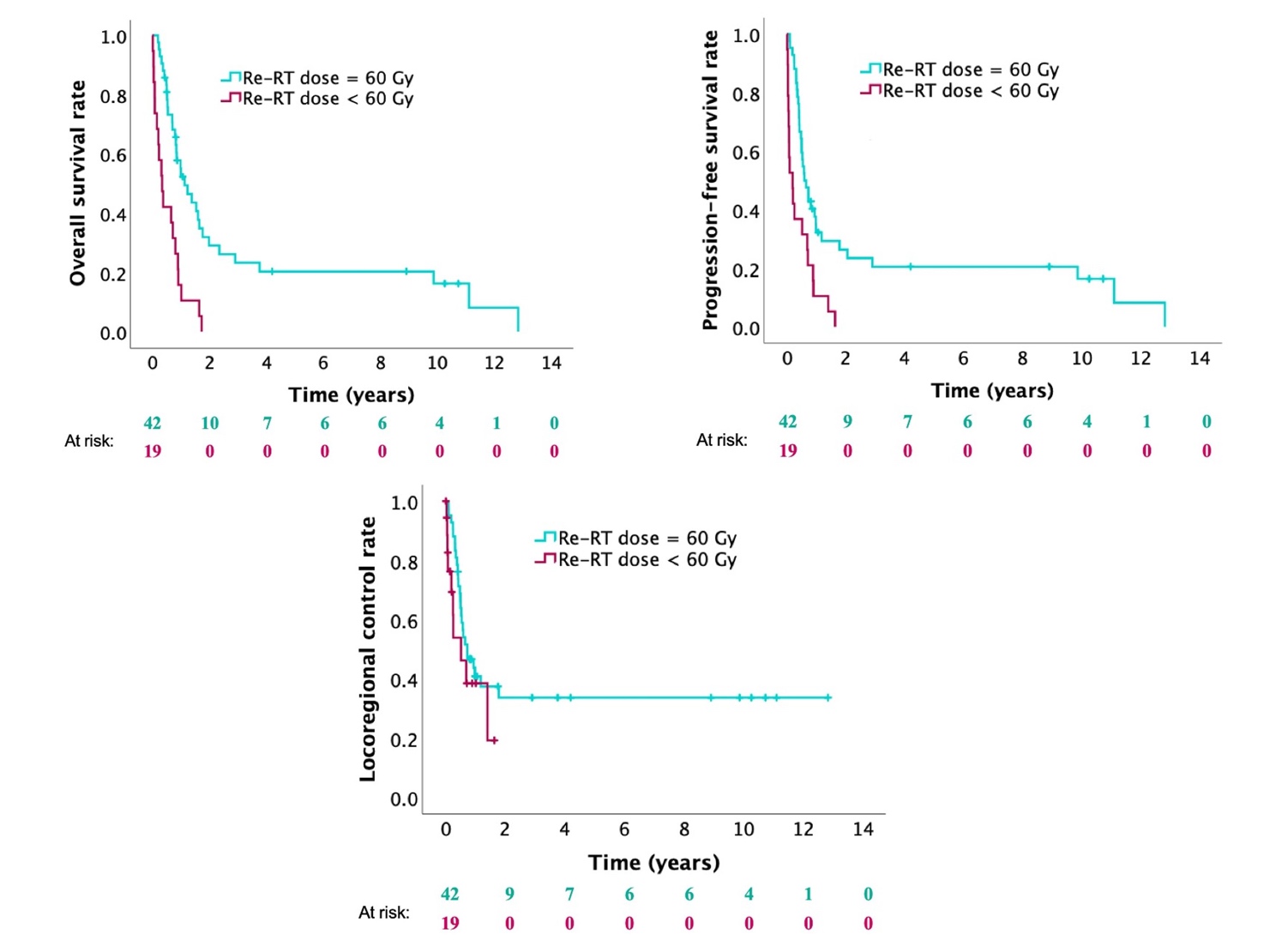


Supplementary Figure 5. Kaplan-Meier curves for (A) overall survival, (B) progression-free survival and (C) locoregional control of patients who received 60 Gy versus patients who received a lower dose.
